# Xylitol enhances synthesis of propionate in the colon via cross-feeding of gut microbiota

**DOI:** 10.1186/s40168-021-01029-6

**Published:** 2021-03-18

**Authors:** Shasha Xiang, Kun Ye, Mian Li, Jian Ying, Huanhuan Wang, Jianzhong Han, Lihua Shi, Jie Xiao, Yubiao Shen, Xiao Feng, Xuan Bao, Yiqing Zheng, Yin Ge, Yalin Zhang, Chang Liu, Jie Chen, Yuewen Chen, Shiyi Tian, Xuan Zhu

**Affiliations:** 1 School of Food Science and Biotechnology, Zhejiang Gongshang University, Hangzhou, 310018 China; 2 Zhejiang Huakang Pharmaceutical Co., Ltd., Kaihua, 324302 China; 3 Nutrition and Health Research Institute, COFCO Ltd., Beijing, 102209 China; 4 School of Medicine, Hangzhou Normal University, Hangzhou, 310018 China; 5 Laboratory of Aging and Cancer Biology of Zhejiang Province, Hangzhou Normal University, Hangzhou, 311121 China; 6 Yangtze Delta Institute of Tsinghua University, Jiaxing, 314000 China; 7 School of Food Science and Technology, Jiangnan University, Wuxi, 214122 China

**Keywords:** Intestinal microorganism, Xylitol, In vitro colonic simulation system (CDMN), Cross-feeding

## Abstract

**Background:**

Xylitol, a white or transparent polyol or sugar alcohol, is digestible by colonic microorganisms and promotes the proliferation of beneficial bacteria and the production of short-chain fatty acids (SCFAs), but the mechanism underlying these effects remains unknown. We studied mice fed with 0%, 2% (2.17 g/kg/day), or 5% (5.42 g/kg/day) (weight/weight) xylitol in their chow for 3 months. In addition to the in vivo digestion experiments in mice, 3% (weight/volume) (0.27 g/kg/day for a human being) xylitol was added to a colon simulation system (CDMN) for 7 days. We performed 16S rRNA sequencing, beneficial metabolism biomarker quantification, metabolome, and metatranscriptome analyses to investigate the prebiotic mechanism of xylitol. The representative bacteria related to xylitol digestion were selected for single cultivation and co-culture of two and three bacteria to explore the microbial digestion and utilization of xylitol in media with glucose, xylitol, mixed carbon sources, or no-carbon sources. Besides, the mechanisms underlying the shift in the microbial composition and SCFAs were explored in molecular contexts.

**Results:**

In both in vivo and in vitro experiments, we found that xylitol did not significantly influence the structure of the gut microbiome. However, it increased all SCFAs, especially propionate in the lumen and butyrate in the mucosa, with a shift in its corresponding bacteria in vitro. Cross-feeding, a relationship in which one organism consumes metabolites excreted by the other, was observed among *Lactobacillus reuteri*, *Bacteroides fragilis*, and *Escherichia coli* in the utilization of xylitol. At the molecular level, we revealed that xylitol dehydrogenase (EC 1.1.1.14), xylulokinase (EC 2.7.1.17), and xylulose phosphate isomerase (EC 5.1.3.1) were key enzymes in xylitol metabolism and were present in *Bacteroides* and *Lachnospiraceae*. Therefore, they are considered keystone bacteria in xylitol digestion. Also, xylitol affected the metabolic pathway of propionate, significantly promoting the transcription of phosphate acetyltransferase (EC 2.3.1.8) in *Bifidobacterium* and increasing the production of propionate.

**Conclusions:**

Our results revealed that those key enzymes for xylitol digestion from different bacteria can together support the growth of micro-ecology, but they also enhanced the concentration of propionate, which lowered pH to restrict relative amounts of *Escherichia* and *Staphylococcus.* Based on the cross-feeding and competition among those bacteria, xylitol can dynamically balance proportions of the gut microbiome to promote enzymes related to xylitol metabolism and SCFAs.

Video Abstract

**Supplementary Information:**

The online version contains supplementary material available at 10.1186/s40168-021-01029-6.

## Introduction

Xylitol is a white or transparent five-carbon polyol or sugar alcohol, which is easily dissolved in water. Xylitol is naturally distributed in fruits, vegetables, and bran but in low fractions [1–3]. In addition, a small amount of xylitol is converted in the human body [4]. Xylitol, known as an indigestible carbohydrate, is of great interest to scientists due to its prebiotic-like characteristics [5]. Several studies have investigated the relationship among the gut microbiota, xylitol, and its related metabolism. Some researches revealed that gut microbiota induced by xylitol inhibit the accumulation of lipids by synthesis of short-chain fatty acids (SCFAs) from prebiotics [6–8]. Moreover, xylitol significantly enhanced the relative amount of phylum *Firmicutes* and genus *Prevotella*, decreasing phylum *Bacteroidetes* and genus *Barnesiella*. Some researchers also found that combined supplementation of glucan and xylitol increased the concentrations of acetate and propionate and decreased the contents of branched-chain fatty acids (BCFAs) with the stability of biogenic amines by gut microbiota [9]. Xylitol also affects the metabolism of daidzein by intestinal microbial or intestinal metabolic activities. In a group of male mice supplied with 0.05% daidzein and 5% xylitol in the diet, the concentration of *Bacteroidetes* was decreased compared with only daidzein supplementation [10]. In addition, a single 30-g oral dose of xylitol fed to human volunteers and rodent shifted both fecal microbiome population from Gram-negative to Gram-positive bacteria [11].

Previous studies have also focused on the roles of gut microbiota involved in the metabolism of xylitol. Some researchers have reported that xylitol and sorbose can significantly promote the production of butyrate in an in vitro fermentation, which was related to an increase in the relative abundance of *Anaerostipes hadrus* and *Anaerostipes caccae* [8]. Only these two bacteria can produce butyrate derived from sorbose and xylitol among 12 typical butyrate-producing bacteria in the colon. However, further studies have found that the pure culture of *Anaerostipes hadrus* DSM 3319 in vitro cannot utilize xylitol [8], indicating that there exists a cross-feeding system between gut bacteria. Moreover, the growth of *Streptococcus pneumoniae* can be inhibited by xylitol to prevent acute otitis media in infants and young children [12, 13], since xylitol cannot be metabolized by it. Xylitol decreased the level of lipopolysaccharide (LPS) on the cell membrane; thus, it inhibits the biofilm formation and growth of bacteria [14, 15]. Many studies also supported this hypothesis [16, 17], but until now, few studies discuss how xylitol alters gut bacteria. On the other hand, researches revealed that xylitol promoted the growth of beneficial bacteria, such as *Bifidobacterium* and *Lactobacillus*, in the rat colon [11, 18]. Based on its characteristics, xylitol is widely and successfully used in the biological medicines and the food industry as a sweetener [19–21]. However, the mechanism underlying the effect of xylitol on gut microbiota and its metabolism remains unknown.

**Fig. 1:**
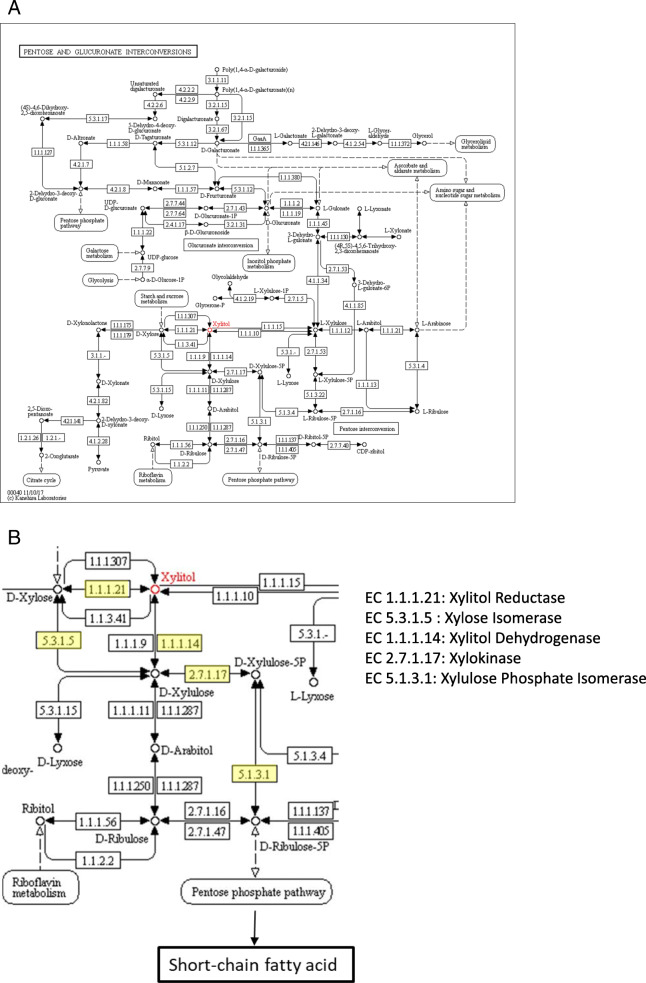
Metabolic pathways of xylitol in KEGG (KEGG: Kyoto Encyclopedia of Genes and Genomes). **A** Overview of the pathways. **B** Known metabolic pathways and enzymes that xylitol is metabolized by microorganisms to produce short-chain fatty acids

A small part of dietary xylitol is absorbed by the small intestine and is metabolized by the normal metabolic pathway of the liver [22]. Over half xylitol is digested in the intestine by bacteria [23]. After xylitol is directly or indirectly converted to D-xylulose, D-xylulose-5P is further converted to fructose-6P in the pentose phosphate pathway. Subsequently, the product enters the tricarboxylic acid cycle to provide energy for cells or to be transformed into other compounds, such as ribose and succinic acid, for the usage of cells. As we know, xylitol can be converted into xylose by microorganisms such as *Escherichia coli* and yeast [24, 25]. Xylose can further be metabolized into pyruvic acid, which is known to be an important intermediate for the metabolism of carbohydrates into short-chain fatty acids as shown in Fig. 1. Xylitol dehydrogenase (EC 1.1.1.14), xylitol oxidase (EC 1.1.3.41), xylitol reductase (EC 1.1.1.21), and xylose isomerase (EC 5.3.1.5) are invertases in two pathways that convert D-xylitol to D-xylulose, respectively. Xylulokinase (EC 2.7.1.17) and xylulose phosphate isomerase (EC 5.1.3.1) are two important enzymes in the pentose phosphate metabolic pathway. However, few studies have reported the enzymes involved in xylitol metabolism during microbiological digestion in the colon till now.

In the previous research [8, 18, 26], xylitol increased the concentrations of SCFAs to benefit human being. As we know, SCFAs have the ability to regulate the uptake of water and minerals, reduce the pH of the colon, inhibit pathogens, and promote the growth of beneficial bacteria [27, 28]. Acetate is produced by most anaerobic bacteria [29] and is responsible for reducing the colonic pH and inhibiting the growth of pathogens. Butyrate, as a primary energy source for the intestinal epithelial cell, has many beneficial functions, such as anti-inflammation and anticancer activities, enhancing the gut barrier, inducing immune cell proliferation and differentiation, and affecting the metabolism of the energy system [6, 30]. Propionate is mainly metabolized in the liver and inhibits cholesterol synthesis after being absorbed [30]. Propionate derived from hexose and pentose are mainly produced by the succinic pathway and can also be produced by the acrylate pathway and the propylene glycol pathway [30–33]. Some researchers demonstrated that propionate treatment in mice with a high-fat diet (HFD) reduced the body weight and systolic blood pressure, lowered fasting insulin levels, and reduced macrovesicular steatosis of the liver, hypertrophy of the liver, and inflammation of the liver induced by HFD [34]. But propionate-treated mice were more anxious in an open field and showed reduced activity of synaptogenesis and glutamate regulators in the hippocampus. However, researchers from Harvard School of Public Health also found that short-term propionate intake from food can cause high blood sugar and insulin. The long-term propionate intake caused more severe symptoms, such as obesity and insulin resistance, but propionate synthesized in the colon does not have these harmful effects [28]. Chambers et al. demonstrated that dietary fibers could prevent weight gain through promoting secretion of PYY and GLP-1 via enhancing the concentration of propionate [35]. Although propionate is beneficial to host health, the depth and comprehensiveness of studies on endogenous propionate synthesis by cross-feeding of gut flora are lacking.

Xylitol can be selectively utilized by microbiota like dietary fibers [36], polysaccharides [37], and other prebiotics [38, 39] to enhance beneficial metabolic pathways. However, few single species of bacteria mentioned above possess the complete genes for xylitol digestion. At present, the regulation of the interaction between xylitol, intestinal flora, and metabolic activity is still unclear. Our research combined in vivo and in vitro methodologies to study this mechanism by evaluating the effects of xylitol, its targeting point, and methods. We want to know how xylitol influences the relative abundance of beneficial bacteria and harmful bacteria. Moreover, we also want to investigate if xylitol changes metabolic activities and pathways after changing the gut microbiota composition. On the other hand, we want to understand how cross-feeding relationships are present among bacteria in the gut, which may be involved in the translation of enzymes. According to microbial metabolic pathways, especially the SCFA pathway, the transcription of related enzymes and keystone bacteria, we can understand the mechanism of the entire flora in the context of individual bacterial species. This study can be considered as a reference for xylitol further studies on the mechanisms of prebiotics and the modulation of the gut microbiota.

## Results

### The shift of gut microbiome in mice treated with xylitol

**Fig. 2:**
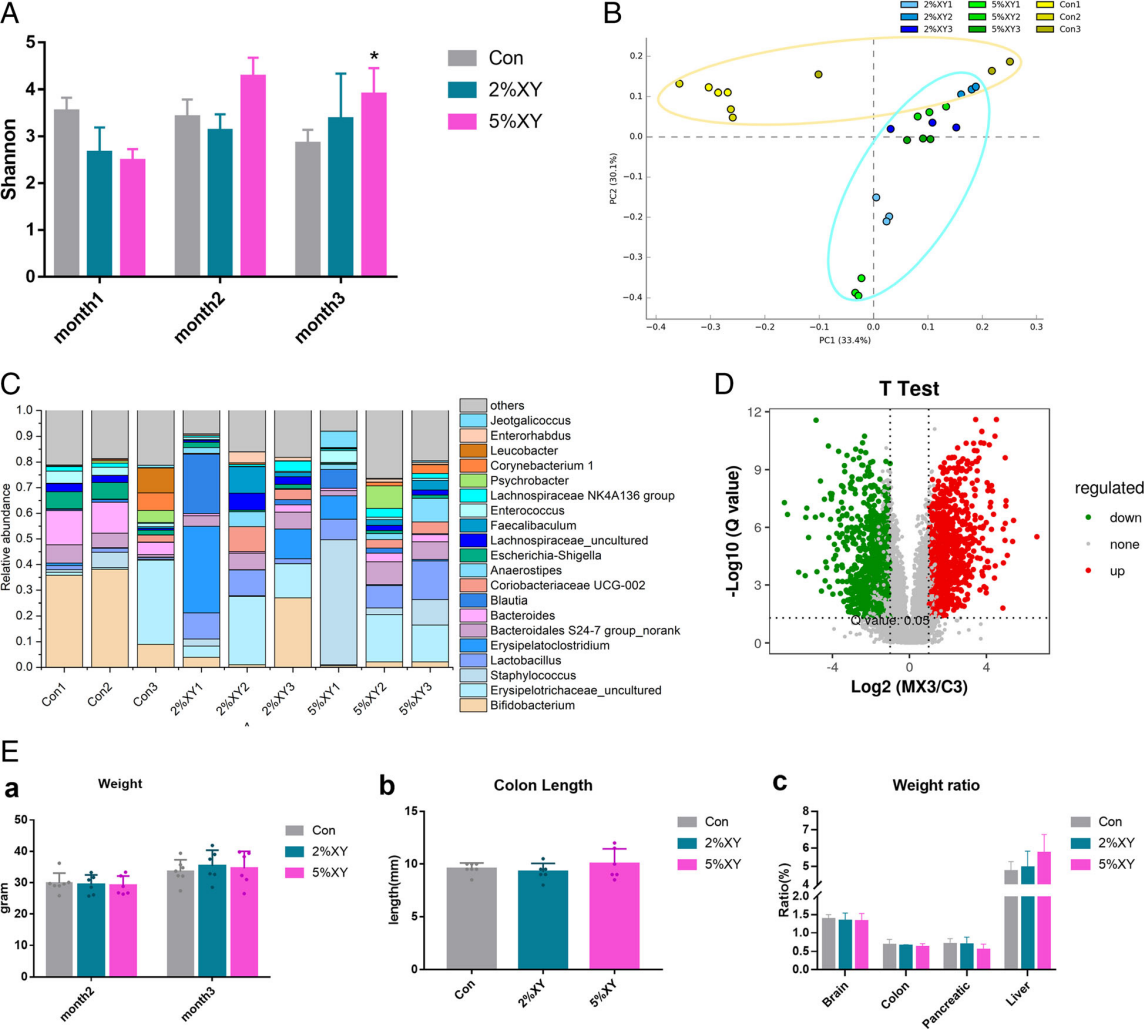
**A** Effects of xylitol on alpha diversity-Shannon index of intestinal microbial in mice. **B** Principal component analysis of intestinal microorganisms (the difference in PC1 and PC2 was 33.4% and 30.1%). **C** Histogram of relative abundance of bacteria in mouse colon (top 20 genera analysis) (*n* = 8 in each group). **D** Volcano map of different metabolite production levels in mice in the third month (*n* = 8 in each group). **E** Effects of xylitol on mouse weight and the weight ratio of the organ. The weight of organs was obtained from the mouse in the third month after sacrifice (*n* = 8 in each group) (In **A**, **B**, **C**, **E**: *Con*, control group; *2%XY*, a cohort of mouse supplied with a diet that contains 2% of xylitol; *5%XY*, a cohort of mice supplied with a diet that contains 5% of xylitol; the number means the month to feed the mice; 1, 2, and 3 represented the first, second, and third months. *p < 0.05. In **D**: red dot—significantly up-produced metabolites, green dot—significantly down-produced metabolites, gray dot—the metabolites with no significant difference *Q* < 0.05)

To determine how xylitol influences gut microbiota in the colon of mice, 8-week-old mice were provided with 0%, 2% (2.17 g/kg/day), or 5% (5.42 g/kg/day) (w/w) xylitol in their chow for 3 months, the doses of which was equal to 0.18 g/kg/day or 0.44 g/kg/day for human being. The stools were collected monthly until sacrifice. A total of 27 samples were collected and sequenced by 16S rRNA in the V4 and V5 regions. After quality control, 565,834 clean reads were obtained. In principle, an average of 47,152 ± 6,338 clean reads per sample with a depth of 420 ± 5 bp, which was an average depth of 99.8 ± 0.8%. After a 97% similarity cluster analysis, 1521 OTUs were obtained. In the evaluation of ecological diversity, alpha diversity can reflect the abundance and diversity of species within a community. The Shannon index is used to indicate the abundance and evenness in a community. A higher Shannon index indicates higher community stability [39]. As shown in Fig. 2A, the Shannon index of the gut microbiota community of mice fed 5% xylitol in chow increased (*p <* 0.05) after 3 months. Meanwhile, the @risk v7.5.0.1 was applied to fit the data and select the optimal distribution according to Akaike’s Information Criterion (AIC) standard. The probability of distribution functions (pdfs) by species under various xylitol supplementation had tiny differences (Figure S1). Moreover, the probability of distribution functions by alpha diversity under various xylitol supplementations was also plotted. The averages of functions were 2.9674, 3.2599, and 3.3154 for 0, 2%, and 5% respectively (Figure S2). Most parts of these functions were overlapped, which means that no big differences were observed after xylitol consumption (Figure S2). All these results indicated that xylitol did not significantly influence the evenness and stability of the colonic community. Principal component analysis (PCA) was introduced for describing the alteration of micro-ecology after xylitol supplementation. In PC1 (33.4%) and PC2 (30.1%) of the two-dimensional diagram, there is a difference between the xylitol group and the control after 1 month. The PCA (Fig. 2B) result indicated that xylitol caused a significant change in the gut microbiota composition.

ANOVA (*T* test) method was used to figure out the influence of xylitol on changing gut bacterial phylum. The relative abundance of bacteria in the colon of mice was shown in Fig. 2C and Figure S3. Xylitol decreased the relative abundance of *Firmicutes* and increased the relative abundance of *Actinobacteria* and *Bacteroidetes* (*p* < 0.05) in the colon of mice with the increasing time. Interestingly, 2% xylitol decreased the relative abundance of *Proteobacteria* (*p* < 0.05) in the second month and increased it in the third month (*p* < 0.05). However, 5% xylitol had the opposite effect. Regardless of increasing dose, *Proteobacteria* continued to dynamically change from15.1% ± 0.1% to 1.4% ± 0.1%.

The false discovery rate (FDR) method was utilized in the comparison of alternation of genera. The composition in the genus level can be found in Fig. 2C. The representative genera in each group were diverse. After 3 months, a huge decrease of *Lactobacillus*, *Staphylococcus*, *Blautia*, and *Anaerostipes* was found in the 2% xylitol supplementation group. The gut microbiota of mice treated with 5% xylitol exhibited a significant increase in the relative abundance of *Bifidobacterium* and *Erysipelotrichaceae*. Notably, a decrease in the relative abundance of *Staphylococcus* (from 48.6% ± 3.2% to 9.8% ± 1.4%) was detected in the third month compared to that in the first month (*Q* < 0.05). The minor change also can be found in a decrease of the relative abundance of *Enterococcus*, *Blautia*, and *Jeotgalicoccus* and an increase of the relative abundance of *Lactobacillus* in the mice treated with 5% xylitol (*Q* < 0.05). Additionally, the relative abundance of *Escherichia-Shigella*, *Lachnospiraceae*, and *Bacteroides* fluctuated in both dosages of xylitol supplementation, while in the control group, we found no significant change in the composition of gut microbiota except *Leucobacter* over 3 months. In conclusion, 5% (5.42 g/kg/day) xylitol increased the relative abundance of *Bifidobacterium*, *Lactobacillus*, and *Erysipelotrichaceae* and decreased the relative abundance of *Blautia and Staphylococcus. Lachnospiraceae* remained stable. Five percent xylitol exhibited the prebiotic effect compared with 2% xylitol and control.

### Changes in the metabolome of mice after consuming xylitol

To understand the influence of xylitol on metabolism, a metabolomic method and SCFA analysis were introduced to figure out the relationship between them. Compared to the control, both concentrations of xylitol promoted the production of acetate (Table S1). In the third month, only 5% xylitol supplementation still increased the acetate concentration from 6.40 ± 1.19 mmol/g to 14.49 ± 0.11 mmol/g with a 2.3-fold increase. This result indicated that 5% xylitol supplementation had a more significant beneficial effect. In the first 2 months, only 5% xylitol supplementation promoted the concentration of propionate (3.23 ± 1.06 mmol/g, 10.02 ± 0.30 mmol/g). Both concentrations of xylitol increased the production of propionate in the third month. Treatment with 5% xylitol increased the propionate concentration to 10.09 ± 0.05 mmol/g (4.6 times higher than the control). Treatment with 2% xylitol did not affect the yield of butyrate, while 5% xylitol had a significant effect on the yields of butyrate (1.14 ± 0.05 mmol/g) and valerate (5.61 ± 0.03 mmol/g). In summary, xylitol significantly increased the total production of SCFAs in the mouse intestine, especially 5% xylitol, which increased the output of acetate, propionate, butyrate, and valerate in the middle and later periods, while 2% xylitol supplementation had no obvious effect on butyrate.

The feces of mice in the group treated with 5% xylitol in chow and the control mice in the third month were chosen for metabolic analyses. There were 8310 features (positive ion feature) detected, including 912 upregulated and 1152 downregulated features (Fig. 2D). The differentially produced metabolites were annotated in the KEGG pathway, and we found that xylitol significantly affected 17 metabolic pathways (*Q <* 0.05), especially the sesquiterpenoid and triterpenoid biosynthesis pathway and the level three pathway of the metabolism of terpenoids and polyketides. Among these pathways, most were involved in amino acid metabolisms, such as lysine degradation, glycine, serine, and threonine metabolism, and tyrosine metabolism analysis.

In conclusion, the in vivo experiments preliminarily evaluated the prebiotic effects of xylitol on the gut microbiota and host by promoting the growth of *Bifidobacterium* and *Lactobacillus*. In addition, xylitol increased the yield of beneficial metabolic pathways, especially propionate. Both metabolomic analysis and SCFA analysis suggested that xylitol mainly affects amino acid metabolism and promotes the concentration of propionate.

### Effect of xylitol consumption on physiological index

Upon sacrifice, the brain, pancreas, colon, and liver were weighed, and the colon length was measured. To reduce the biological difference, the weight ratio of organs was calculated by organ weight divided by mouse weight. As shown in Fig. 2E, there were no significant effects (*p* < 0.05) observed with 2% (2.17 g/kg/day) or 5% (5.42 g/kg/day) xylitol. These results indicated that xylitol supplementation had no significant effect on the physiology of mice during this study.

### Alternation of the gut microbiome in an in vitro simulation system with xylitol supplementation

To determine whether a cross-feeding system exists in gut microbiota from human beings during xylitol digestion, a CDMN system was used to investigate the bacterial composition and metabolism in an in vitro gut lumen and mucosa. In our study, 3% weight/volume of xylitol was utilized according to FDA instruction (Guidance for Industry Estimating the Maximum Safe Starting Dose in Initial Clinical Trials for Therapeutics in Adult Healthy Volunteers), which is equal to 0.27 g/kg/day for a human and which is approaching to the daily recommended intake amount of xylitol. In the 1970s, the Joint FAO/WHO Expert Committee on Food Additives (JECFA) recognized xylitol as a safe food without ADI values and the industry recommended that the daily intake should not exceed 40 g. The CDMN was chosen to study the dynamic change of gut microbiota with the addition of xylitol, because its bacterial composition is high similar to human feces. In the gut lumen (Figure S4A), after being supplied with xylitol, the relative abundance of phyla in the gut microbiota changed substantially. We can see that in the ascending colon (AC), xylitol increased the relative abundance of *Firmicutes*, while it decreased the relative abundance of *Synergistetes* and *Proteobacteria*. An increase in the relative abundance of *Firmicutes* and a decrease in the relative abundance of *Bacteroidetes* and *Proteobacteria* were detected in the transverse colon (TC). In the descending colon (DC), the relative abundance of *Firmicutes* increased, while that of *Proteobacteria* decreased (*p* < 0.05). In summary, xylitol increased the relative abundance of *Firmicutes*, while it decreased that of *Proteobacteria* in the gut lumen, which was similar to the results of mice.

**Fig. 3:**
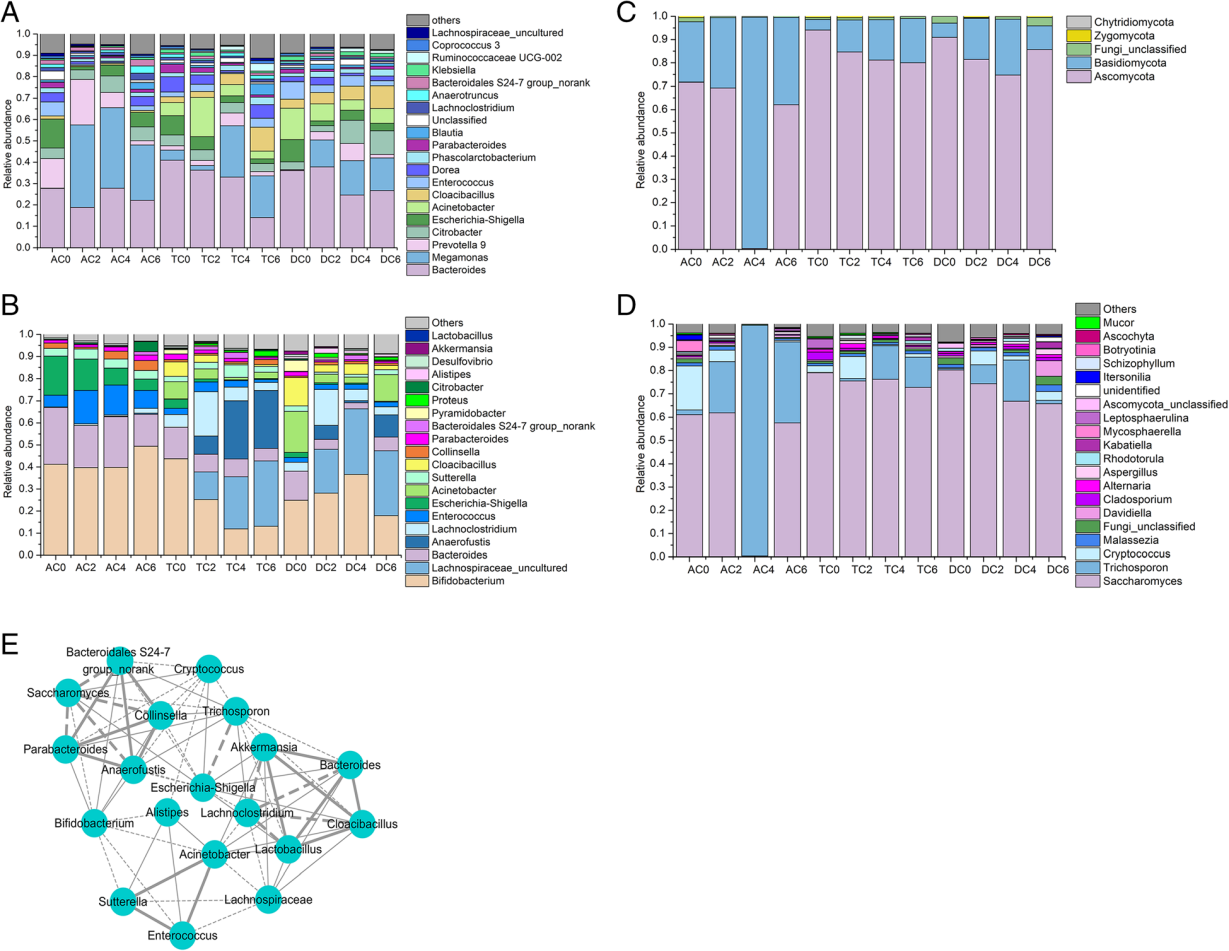
**A** Histogram of relative abundance of bacteria in different parts of in vitro colon (top 20 genera of bacteria in the lumen). **B** Histogram of relative abundance of bacteria in different parts of in vitro colon (top 20 genera of bacteria in the mucosa). **C** Histogram of relative abundance of fungi in different regions of the colon (phylum). **D** Histogram of relative abundance of fungi in different regions of the colon (top 20 genera of fungi in the lumen). **E** Spearman correlation analysis of microorganisms (solid line—positive correlation, dotted line—negative correlation, thick line—significant correlation *p <* 0.01. AC, TC, and DC presented the ascending colon, transverse colon, and descending colon; the number means the days for adding xylitol)

The FDR method was also applied in this experiment. The results of the top 20 most abundant genera in the gut microbiota of the gut lumen are shown in Fig. 3a. The predominant bacteria belong to the genus *Bacteroides*, *Megamonas*, *Prevotella 9*, *Escherichia*, and *Enterococcus*. The community structures of AC and DC were the most unstable after xylitol stimulation, and the relative abundance of bacteria in each genus changed greatly, which indicated that xylitol affected these two regions. Supplementation with xylitol induced a decrease in the relative abundance of *Escherichia-Shigella* in the ascending colon (*Q* < 0.05) and an increase in that of *Megamonas* (*Q* < 0.05). The microbial community structure in the transverse colon was relatively stable. Only a slight increase in the relative abundance of *Blautia* was detected on the 6th day. The decrease in the abundance of *Escherichia-Shigella*, *Enterococcus*, and *Coprococcus* was observed in the descending colon (*Q* < 0.05). Xylitol did not shift the abundance of *Bacteroidetes,* which was the dominated genus in all regions of the colon. Overall, xylitol decreased the proportion of *Escherichia-Shigell* and *Enterococcus* in CDMN. Surprisingly, the composition of *Lactobacillus* and *Bifidobacterium* was stable, which are in theory can easily be affected by xylitol.

There was some composition shifted in the bacteria in the gut mucosa when xylitol was added into CDMN (Figure S4B). In the ascending colon, the relative abundance of *Actinobacteria* and *Firmicutes* increased, while that of *Proteobacteria* decreased. No significant change was detected in the abundance of *Bacteroidetes*. In the transverse colon, the fraction of *Firmicutes* increased, while that of *Actinobacteria* and *Proteobacteria* decreased, with no significant difference in that of *Bacteroidetes*. An increase in the relative abundance of *Actinobacteria* and *Firmicutes* and a decrease in the relative abundance of *Bacteroidetes* and *Proteobacteria* were detected in the descending colon. The above significant value was *p* < 0.05. The top 20 genera in the gut mucosa are shown in Fig. 3b and the FDR method was also applied. *Bifidobacterium*, *Lachnospiraceae*, *Bacteroides*, *Anaerofustis*, and *Enterococcus* were the dominant genera in the mucosal layer. The most unstable community structure in the mucosa was found in the transverse colon and descending colon, indicating that xylitol had a high influence on microbiome in the mucosa of the large intestine. Xylitol increased the fraction of *Parabacteroides*, *Bacteroidales S24-7 group_norank*, *Lachnoclostridium*, *Citrobacter*, and *Anaerofustis* in the mucosa of the ascending colon. While the relative abundance of *Escherichia-Shigella* and *Desulfovibrio* decreased in the same region on the last day. Xylitol had more effects on the transverse colon and descending colon. After 6-day supplementation, a significant decrease in the relative abundance of *Bifidobacterium*, *Escherichia-Shigella*, *Enterococcus*, *Desulfovibrio*, and *Alistipes* was detected and a huge increase can be observed in the relative abundance of *Proteus*, *Citrobacter*, *Bacteroidales* S24-7 group_norank, *Anaerofustis*, and *Lachnospiraceae*. In the descending colon, the dramatic change of microbial community can be found on the 4th day. The relative abundance of *Bifidobacterium*, *Lachnoclostridium*, *Lachnospiraceae*, and *Anaerofustis* were enriched, while the relative abundance of *Bacteroides* and *Escherichia-Shigella* were decreased. There was no significant difference in the change of the relative abundance of *Lactobacillus* in the three regions of the colon. In general, xylitol increased the fraction of *Lachnospiraceae* and decreased that of *Escherichia-Shigella* in each intestinal region.

As we know fungi were usually involved in the metabolism of xylitol, the ITS method was used to investigate the relationship between xylitol and fungi. *Ascomycota* and *Basidiomycota* accounted for 95% of the fungi in the human gut. More than 60% of fungi were members of the *Ascomycota* phylum (Fig. 3c) and represented by *Saccharomyces.* Xylitol mainly altered the fungi composition in AC, especially on the 6th day. The relative abundance of *Saccharomyces*, *Malassezia*, *Cryptococcus*, *Cladosporium*, and *Botryotinia* was higher on the 6th day than that on the 4th day, while a huge decrease of the relative abundance of *Trichosporon* was observed, decreasing from 99.12 to 34.67% (*Q* < 0.05). Xylitol also induced the composition alteration in TC. An increase in the relative abundance of *Cryptococcus*, *Wallemia*, and *Eutypa* and a decrease in the fraction of *Mucor* and *Microascus* were detected on the 6th day compared to that on the 4th day (*Q* < 0.05). No significant change can be found in TC. In conclusion, xylitol decreased the relative abundance of *Saccharomyces.* In contrast, xylitol increased the fraction of *Trichosporon* (Fig. 3d). The human commensal *Trichosporon* spp. can adapt to the host microenvironment and its immune system [40].

After analysis of the Spearman correlation among microbiota, the relationship and interaction among gut microbiota were determined, as shown in Fig. 3e. There were three significant interaction networks. *Saccharomyces* negatively modulated *Bacteroidales S24-7 group_norank*, *Collinsella*, *Parabacteroides*, and *Anaerofustis* in the network. *Lachnoclostridium* negatively modulated *Akkermansia*, *Lactobacillus*, *Bacteroides*, and *Cloacibacillus.* There was a positive network among *Acinetobacter*, *Sutterella*, and *Enterococcus*. Although *Bifidobacterium* did not join any of the three significant interaction networks, it had a close relationship among them. *Bifidobacterium* was associated with three bacterial interaction networks of *Saccharomyces* and *Acinetobacter*. In addition, it was also associated with the network of *Lachnoclostridium* by connection with *Anaerofustis*. *Bifidobacterium* negatively correlated with the network of *Lachnoclostridium* and *Acinetobacter*. *Escherichia-Shigella* had a positive correlation with *Lactobacillus* and *Bacteroides*. Regardless of the pattern by which *Escherichia-Shigella* interacted with other bacteria, *Bifidobacterium* always inhibited it. In addition, *Bifidobacterium* was negatively correlated with the SCFA-producing bacteria *Lachnospiraceae* and *Anaerofustis*. Those results indicated that several genera of bacteria or fungi were involved in xylitol metabolism by a cross-feeding system. Regarding Spearman analysis, the whole network of microbiota was divided into several parts, in which genera *Escherichia-Shigella*, *Lactobacillus*, *Bifidobacterium*, and *Bacteroides* dominated key positions and had close interactions with many bacteria in the community.

### Changes of metabolome induced by xylitol in an in vitro simulation

In order to investigate the alternation of the metabolome, an electronic sensor was introduced to examine the alterations of gases from CDMN during the experiment. Ammonia, hydrogen sulfide, hydrogen, and methane are the primary components of intestinal gases (Figure S5), which were converted from different nutrients in the intestinal medium. The gas analysis can preliminarily reflect the metabolism of the gut microbiota. There was no significant difference in the gas composition in the ascending colon compared with the baseline level of gas. The sulfide (SE2), hydrogen (SE3), and methane (SE14) in the transverse colon decreased. Less microbial ammonia (SE1) was produced in the descending colon.

**Fig. 4:**
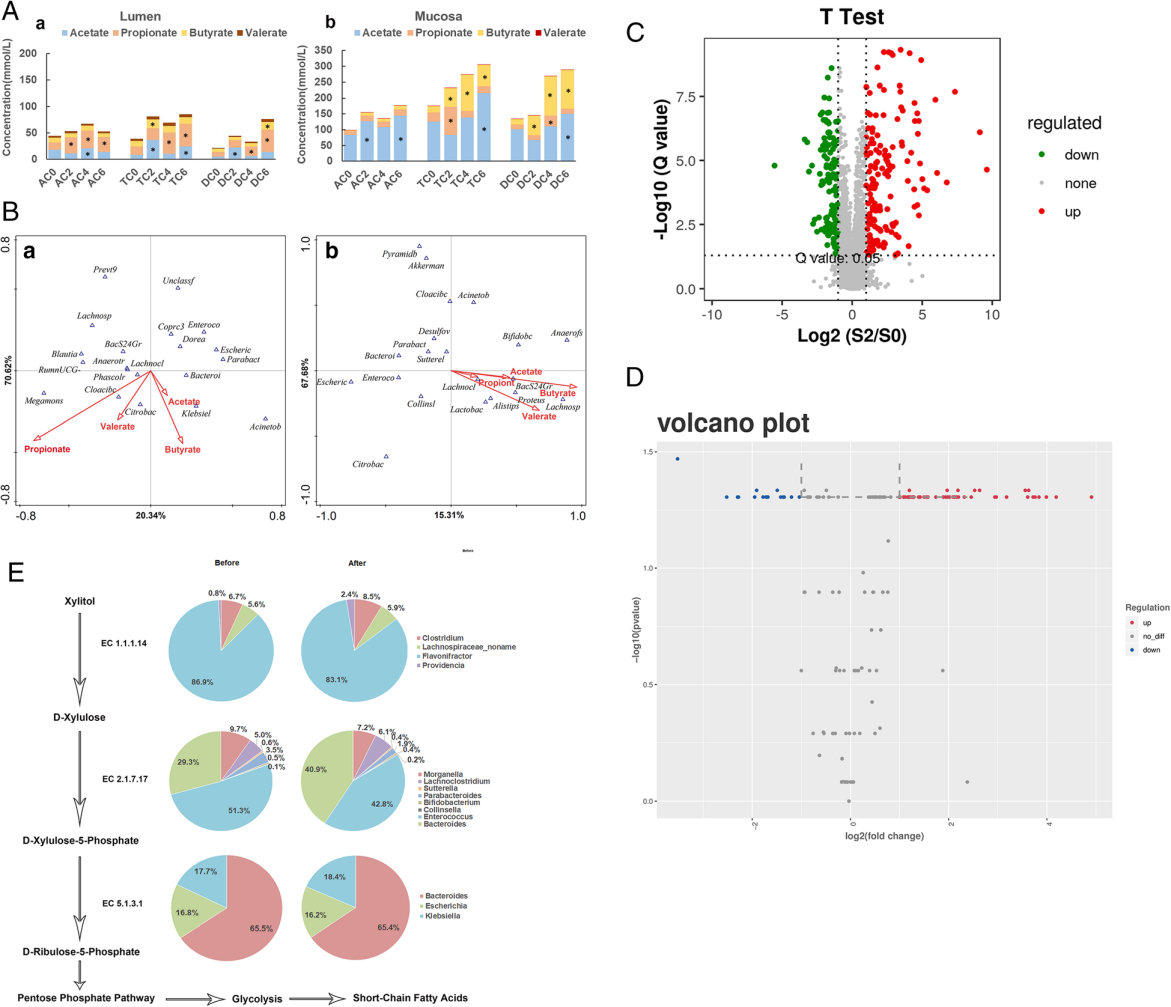
**A** Figure histogram of changes in SCFAs in the gut lumen and mucosa of the colon (*p < 0.05 compared with day 0; AC, TC, and DC presented the ascending colon, transverse colon, and descending colon; the number means the days for adding xylitol; day 0 presented the sample before adding xylitol). **B** Canonical correlation analysis (CCA) between species and short-chain fatty acids ((a) lumen, (b) mucosa. The angle of bacteria and SCFAs less than 90° indicated they are in correlation, but not for more than 90°. The length of the arrow represents the impact on community). **C** Volcano map of different metabolite production levels in vitro (red dot—significantly up-produced metabolites, green dot—significantly down-produced metabolites, gray dot—the metabolites with no significant difference, *Q* < 0.05). **D** Volcano map of different gene expression levels (red dot—significantly upregulated genes, blue dot—significantly downregulated genes, gray dot—the genes with no significant difference, *Q* < 0.05). **E** Distribution of xylitol metabolic enzymes in microorganisms before and after xylitol supplementation (EC 1.1.1.14-xylitol dehydrogenase; EC 2.7.1.17-xylokinase; EC 5.1.3.1-xylose phosphate isomerase. Before-AC0, the sample in the ascending colon before adding xylitol was defined as Before; After-AC2, the sample in the ascending colon after adding xylitol for 2 days was defined as After)

In addition, the SCFA analysis was also applied to investigate the relationship between xylitol and microbiota. The concentration of SCFAs in the mucosa was twice higher than that in the lumen (Fig. 4A). Xylitol significantly increased the yield of SCFAs both in the lumen and mucosa. However, the types and distribution of SCFAs varied. The concentrations of propionate in the colon and butyrate in the mucosal layer were increased by xylitol. On the 6th day, compared to the baseline level, the propionate level increased by 2.1 folds from 13.6 ± 1.5 mmol/L to 28.4 ± 2.1 mmol/L in the lumen of the ascending colon. In the mucosa of the transverse colon, the yield of butyrate increased from 19.5 ± 3.4 mmol/L to 68.5 ± 4.5 mmol/L. The concentration of propionate in the lumen increased to 43.1 ± 2.3 mmol/L, and the concentration of butyrate in the mucosa of the transverse colon increased to 113.0 ± 5.6 mmol/L in the treated group compared to the data at *T* = 0 (time). In conclusion, the *in-site* propionate was synthesized from xylitol by gut microbiota. Therefore, it is mainly produced from the microbiota in the mucosa for the rapid utilization.

Canonical correlation analysis (CCA) is a statistical analysis that comprehensively describes the correlation between two variables. In this study, the correlation between the relative abundance of the gut microbiota and the concentration of SCFAs was used for CCA, which can attribute the production of SCFAs to species. As shown in Fig. 4B (a), propionate and valerate were distributed in the third quadrant, while acetate and butyrate were distributed in the fourth quadrant. Most microbiota was in the first, second, and third quadrants. The angle between propionate and almost all microorganisms in the second and third quadrants was sharp. This result indicated that many bacteria, such as *Lachnospiraceae*, *Megamonas*, *Lachnoclostridium*, and *Cloacibacillus*, were involved in the production of propionate. However, only limited bacteria contributed to the production of butyrate. In the mucosal layer (Fig. 4B (b)), SCFAs were distributed in the fourth quadrant, and the butyrate production in the mucosa was the highest, which is mainly related to the microorganisms in the first and second quadrants, including *Bifidobacterium*, *Anaerofustis*, *Sutterella*, *Bacteroidales S24-7 group_norank*, and *Citrobacter* in addition to the reduced relative abundance of *Escherichia-Shigella*. Interestingly, in either the lumen or mucosa, *Escherichia-Shigella* and *Enterococcus* were not associated with the production of SCFAs, which was in accordance with the results of mice

### Metabolomic profile of samples in an in vitro simulation

From the results of the gut microbiota composition, the community in the ascending colon shifted substantially, especially on the beginning day and 2nd day. Therefore, the metabolomic profiles of these samples were selected for analysis. There were 8310 features detected, including 161 upregulated and 145 downregulated features (Fig. 4C). In total, 17 pathways showed significant differences with the addition of xylitol (*Q* < 0.05). Most of them were catalogized into the secondary pathway of amino acid metabolism and metabolism of other amino acids (5 tertiary pathways) and carbohydrate metabolism (7 tertiary pathways). Although there was no significant difference observed in the propionate pathway (*Q* = 0.0596), we found that metabolites such as methylglyoxal, 2-methyl-cis-aconitate, and 2-methyl-trans-aconitate were enriched (Figure S6). For pentose and glucuronate interconversion (*Q* = 0.167), the production of downstream products of xylitol conversion, such as D-arabitol and ribitol, were enhanced (Figure S7). The results of xylitol digestion pathway analysis from the metabolome coincided with those of the metatranscriptome.

### Effect of xylitol on the metatranscriptome of colonic microorganisms

**Fig. 5:**
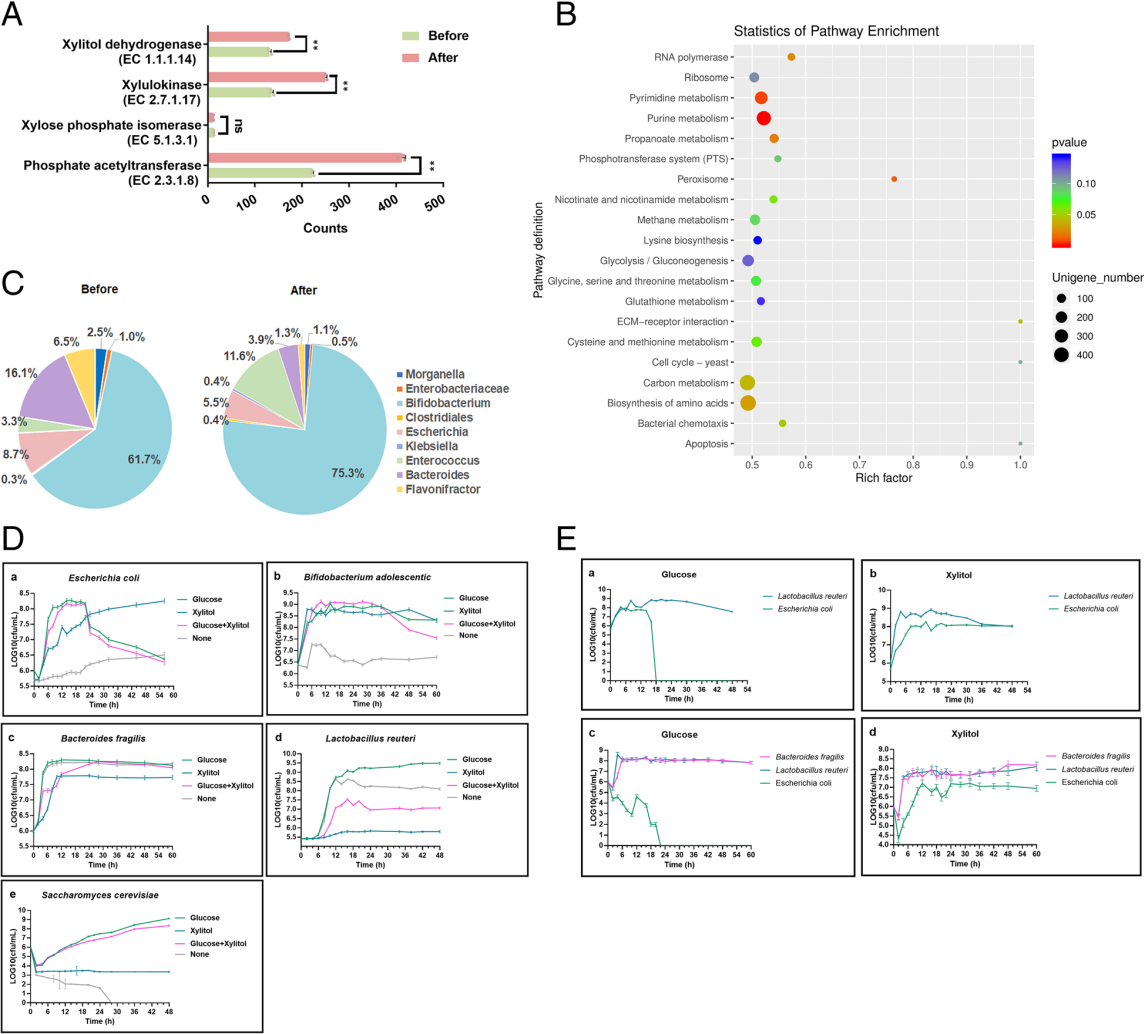
**A** The quantity of enzyme before and after xylitol supplementation (Before-AC0, After-AC2). **B** Scatter plot of KEGG enrichment of differentially expressed genes (Rich factor: the number of differential genes located in the KEGG. The larger the rich factor is, the higher the concentration of KEGG is. Color: metabolic significance difference value). **C** Distribution of acyl phosphate transferase (EC 2.3.1.8), a key enzyme for propionate metabolism, in microorganisms (Before-AC0, After-AC2). **D** Growth curve of intestinal microorganisms in different carbon sources ((a) *Escherichia coli*, (b) *Bifidobacterium adolescentis*, (c) *Bacteroides fragilis*, (d) *Lactobacillus reuteri*, (e) *Saccharomyces cerevisiae*). **E** Growth curve of co-cultivation of double and three bacteria ((a) glucose as the carbon source in double bacterial cultivation; (b) xylitol as the carbon source in double bacterial cultivation; (c) glucose as the carbon source in three bacterial cultivation; (d) xylitol as the carbon source in three bacterial cultivation) (Before-AC0, the sample in the ascending colon before adding xylitol was defined as Before; After-AC2, the sample in the ascending colon after adding xylitol for 2 days was defined as After)

In consistence with metabolomic analysis, the same samples were selected and collected for metatranscriptome analysis. The effect of xylitol on the transcriptome of colonic microorganisms is shown in Fig. 4. The differentially expressed genes after transcriptomic sequencing were annotated by GO. The distribution of differentially expressed genes was presented in a volcano map in Fig. 4D. In total, 29,224 types of genes were detected and 12,926 types of genes showed significant changes in this study, including 8930 upregulated genes (red) and 3996 downregulated genes (blue). After gene sequencing, assembly, and annotation, the distribution of enzymes based on xylitol digestion in the microbiota is shown in Fig. 4E. The quantity of enzyme related with xylitol were all increased after xylitol supplementation (Fig. 5A). Xylitol dehydrogenase (EC 1.1.1.14), xylulokinase (EC 2.7.1.17), and xylose phosphate isomerase (EC 5.1.3.1) were the basic digestive enzymes of xylitol, and the microorganisms that can express these enzymes are considered the basis of xylitol digestion in bacteria. The distribution of xylulokinase (EC 2.7.1.17) in intestinal microorganisms was the most abundant, followed by xylitol dehydrogenase (EC 1.1.1.14) and xylose phosphate isomerase (EC 5.1.3.1). However, not all enzymes exist in a single bacterium. For example, xylitol dehydrogenase (EC 1.1.1.14) was mainly distributed in *Flavonifractor*, *Clostridium*, *Providencia*, and *Lachnospiraceae*. Among them, only *Lachnoclostridium* had xylulokinase (EC 2.7.1.17). In the pie chart of the distribution of xylulose phosphate isomerase (EC 5.1.3.1), no bacteria were found in the family of *Lachnospiraceae*. We suggested that the microorganism with the enzyme xylitol dehydrogenase (EC 1.1.1.14) transformed xylitol into xylose and transferred xylose to the microorganism containing xylulokinase (EC 2.7.1.17). The metabolites were further transferred to the bacteria with xylose phosphate isomerase (EC 5.1.3.1) for gradual utilization. This result further proved that there was a cross-feeding system in the microbiota related to xylitol digestion. After the ingestion of xylitol, the distribution of enzymes in microbiota also changed. The proportion of xylitol dehydrogenase (EC 1.1.1.14) in *Flavonifractor* decreased from 87 to 83%. In addition, the fraction of *Lachnospiraceae*, *Clostridium*, and *Providencia* increased. The proportion of xylulokinase (EC 2.7.1.17) in *Bacteroides* and *Lachnoclostridium* increased from 29% and 5% to 41% and 6%, respectively. The change in the distribution of xylulose phosphate isomerase (EC 5.1.3.1) was limited, with only a 1% difference between *Escherichia* and *Bacteroides.* Xylitol dehydrogenase (EC 1.1.1.14) and xylulokinase (EC 2.7.1.17) were likely two key enzymes in the microbial digestion of xylitol, while *Lachnospiraceae* bacteria also had the ability to translate these two enzymes, as key enzymes for bacterial xylitol digestion. *Bacteroides* was also important in xylitol digestion because it had the ability to translate xylulokinase (EC 2.7.1.17) and xylulose phosphate isomerase (EC 5.1.3.1).

Enrichment analysis of the functions of differentially expressed genes can reveal the function of microorganisms. The pathway enrichment analysis was performed based on the KEGG database. The significantly enriched pathways are shown in Fig. 5B. We found 9 pathways with significant differences (*p* < 0.05) in propionate, purine, and carbohydrate metabolism. Amino acid synthesis was the most abundant and significantly changed pathway. The results showed that xylitol digestion not only changed the composition of the gut microbiota, but also affected the metabolic pathways of other nutrients.

Xylitol promoted the production of propionate, upregulating 66 genes and downregulating 31 genes, including the upregulation of the transcription of a limiting enzyme associated with propionate synthesis in the succinic acid pathway (Figure S8). There were 2 enhanced pathways from propionyl-CoA to propionate. One pathway required 2 steps for phosphorylation, while the other required one step for direct conversion. Phosphate acetyltransferase (EC 2.3.1.8) was the limiting enzyme of propionate metabolism, which was mainly distributed in *Bifidobacterium*, *Bacteroides*, *Escherichia*, and *Enterococcus* (Fig. 5C). The quantity of this enzyme was increased (Fig. 5A) in this study.

Xylitol induced a shift in the enzyme distribution in microorganisms. The proportions of enzymes in *Bifidobacterium* and *Enterococcus* increased from 62% and 3% to 75% and 8%, respectively, with decreasing in the proportions of enzymes in other bacteria, especially *Escherichia* and *Bacteroides*, which was similar to the relative abundance of these bacteria in the gut. Therefore, *Bifidobacterium* and *Enterococcus* were potential key bacteria that promoted the production of propionate. However, from the results of CCA, we knew that *Enterococcus* had no relationship with SCFA production. In conclusion, *Bifidobacterium* was vital for propionate production. Propionate CoA-transferase (EC 2.8.3.1), which functioned in the one-step pathway, was present only in *Flavonifracto*r. The transcriptomes were upregulated after the ingestion of xylitol in the experiments, indicating that *Flavonifracto*r mainly converted propionyl-CoA to propionate with the help of propionate CoA-transferase. Our results revealed that those key enzymes for xylitol digestion from different bacteria can together support the growth of micro-ecology, but they also enhanced the concentration of propionate, which lowered pH to restrict relative amounts of *Bacteroides* and *Escherichia.* Based on the cross-feeding and competition between those bacteria*,* xylitol can dynamically balance proportions of the gut microbiome to promote relative abundances of beneficial bacteria and SCFAs.

### Xylitol as a carbon source for single-strain cultivated and co-cultivated bacteria

Emerging evidence indicated the saccharides or prebiotic can be used by colonic bacteria solely or by cross-feeding among two or several bacteria [41]. To confirm whether xylitol can be used by single bacteria or not, the single-strain cultivation and co-cultivation methods were introduced. Gut bacteria were selected according to the network made by Spearman analysis, which showed the importance of bacteria in the gut microbiota for xylitol digestion. The basic nitrogen medium was prepared with or without the sole carbon sources of glucose and xylitol or the combination of glucose and xylitol for the cultivation of *Escherichia coli*, *Bifidobacterium adolescentis*, *Bacteroides fragilis*, *Lactobacillus reuteri*, and *Saccharomyces cerevisiae*. As we know, *Lactobacillus reuteri* and *Saccharomyces cerevisiae* did not contain enzymes related to xylitol metabolisms. The results of xylitol consumption are shown in Fig. 5D. There was no difference in the growth of *E. coli* in any of the four media at the early stage. However, the number of *E. coli* in the medium with glucose as the carbon source started to decline at 20 h, but this trend was not observed for the other two media. This result illustrated that *E. coli* had the capacity to use carbon and nitrogen sources. In addition, because *E. coli* was also able to use xylitol for growth to stabilize its biomass, no rapid decline was observed in the medium with a complex carbon source. Compared with the negative control, *B. adolescentis* grew on medium with only xylitol and showed no significant difference in growth on the other media (Fig. 5D (b)). These results showed that xylitol can be used by *B. adolescentis* as a carbon source. Although *B. fragilis* survived on the xylitol medium, it did not grow as well as it did on the media with glucose or without a carbon source. It means that xylitol inhibited the growth of *B. fragilis* (Fig. 5D (c)). *L. reuteri* had limited growth on the xylitol medium. Beyond our expectation, *L. reuteri* with a combination supplementation of glucose and xylitol showed a low growth rate in the first 30 h, compared with glucose supplementation. Therefore, xylitol cannot be directly used by *L. reuteri* and further influenced the carbon metabolism of *L. reuteri* (Fig. 5D (d)). Although it rapidly declined on the medium lacking a carbon source, *S. cerevisiae* grew weakly in xylitol medium, but far less than it did on glucose as a carbon source, which showed that *S. cerevisiae* uses xylitol weakly (Fig. 5D (e)).

The growth of bacteria in co-cultivation medium is shown in Fig. 5E. *L. reuteri* and *E. coli* were grown in a medium containing glucose. However, the growth of *E. coli* was inhibited in the later period because of the acidification of the medium by *L. reuteri* (Fig. 5E (a)). Interestingly, on xylitol medium, we found that *L. reuteri*, which could not use xylitol in the single-strain cultivation, grew well and that the number of *L. reuteri* was much higher than the number of *E. coli* (Fig. 5E (b)). This result indicated that *E. coli* can support *L. reuteri* to use xylitol without much acidification. The growth situation in the co-cultivation of three organisms was similar to that in the co-culture of two species. On glucose medium, *L. reuteri* also inhibited the growth of *E. coli* by acid production but had no effect on *B. fragilis*. The growth rate of *E. coli* was always lower than that of *L. reuteri* and *B. fragilis*. This indicates that *L. reuteri* and *B. fragilis* were dominant bacteria, but xylitol had no effect on the community structure (Fig. 5E). In conclusion, a cross-feeding system involved into the utilization of xylitol by gut microbiota.

## Discussion

Understanding how xylitol works as a prebiotic for human beings is critical in xylitol application. In this study, we evaluated the mechanism by which xylitol influenced the gut microbiota, metabolic activity, and beneficial metabolism markers in vivo and in vitro. Collectively, according to the microbiota, metabolome, and metatranscriptome results, we revealed the mechanism of the effect of xylitol on propionate synthesis at the molecular level.

As a result, probability distribution functions (pdfs) of relative species data (RSD) indicated that no great difference between various concentrations of xylitol supplementations was found. Meanwhile, the pdfs of alpha diversity also demonstrated that xylitol had little influence on the diversity of the microbiome. Both results exhibited a no-linear effect of xylitol on the microbiome, which was similar with the results of Lijie [42]. Lijie et al. stated that those profiles of pdfs can be used to identify the situation of the microbiome. In this case, we found that xylitol had no influence on the composition of micro-ecology and further little influenced on the physiology of mice, which was also supported by our experiment. As a result, some bacteria should play a key role in xylitol metabolism. In the mouse experiments, we found that 5% (0.44 g/kg/day for a human being) xylitol in chow promoted the evenness and maintained the stability of the micro-ecology in the gut. However, a difference was detected in the 5% xylitol in the chow treatment group. The consumption of 5% xylitol increased the relative abundance of *Bifidobacterium* and *Lactobacillus*, maintained the concentrations of *Bacteroides* and *Lachnospiraceae*, and decreased the relative abundance of *Escherichia* and *Staphylococcus* (Fig. 2D). However, a high relative amount of *Staphylococcus* was found in the group of 0.44 g/kg/day (for human being) xylitol in chow in the first month. The genus *Staphylococcus* contains not only pathogen *Staphylococcus aureus*, but also symbiotic *Staphylococcus carnosus*, which can be used as a start culture for sausage fermentation [43]. Until sacrifice, we did not find significant differences in the physiology of mice fed either 2% (0.18 g/kg/day for a human being) or 5% xylitol in their chow, indicating that a novel stability of gut-ecology was formed through propionate synthesis after a long-term xylitol supplementation. Although microbiota in mice are varied with that in human beings, their advantages are extensive and the breadth and depth of research on mouse gastroenterology, immunology, and genetics largely exceed any other model [44, 45]. A reasonable diet, suitable species, and stable environment factors can enhance the similarity and repeatability of the human gut microbiota shifts in mice. Mouse experiments of xylitol were used to investigate potential xylitol mechanisms and their interplay between host, microbial, and environmental factors. Considering the in vivo results and calculations according to FDA introduction (Guidance for Industry Estimating the Maximum Safe Starting Dose in Initial Clinical Trials for Therapeutics in Adult Healthy Volunteers), 3% xylitol (0.27 g/kg/day for a human being) was applied in an in vitro experiment to investigate microbial digestion of xylitol in microbiota from a human being colonic simulation. In general, xylitol maintained the fraction of *Lactobacillus* and *Bifidobacterium*, while it decreased that of *Escherichia-Shigella* in each intestinal region, and the relative amount of *Lachnospiraceae* was increased in the mucosal layer (Fig. 3a, b). Among them, *Bifidobacterium* and *Lactobacillus* are the most well-known beneficial bacteria in the gut. The genus *Bifidobacterium* contains 80 species, which are distributed across different niches and an important bacterium for propionate synthesis, including the human gastrointestinal tract. *Bifidobacterium* rapidly becomes the dominant bacteria in the infant’s gut after birth because of milk-derived oligosaccharides (human milk oligosaccharides) that simulate the proliferation of *Bifidobacterium* [46]. *Lactobacillus* species, such as *L. reuteri*, *L. plantarum*, and *L. rhamnosus*, are widely used as probiotics to prevent gut dysbiosis [47], although they do not produce propionate. But, the selective stimulation of *Bifidobacterium* and *Lactobacillus* by prebiotics has been widely shown, and *Bifidobacterium* and *Lactobacillus* are important biomarkers for healthy gut micro-ecosystems [48, 49]. Those bacteria were also reported to support the growth of each other [50]. The selective enhancement of these two genera of beneficial bacteria is considered to be the reason why prebiotic therapies could be beneficial for children who have developed attention deficit hyperactivity disorder [51]. *Lachnospiraceae*, which was changed significantly in our study, is a family of *Clostridia*, some species of which have the capacity to produce butyrate in the gut. Conor et al. analyzed the genome of 30 sequenced *Lachnospiraceae* and found that only 12 of them had the gene annotated from at least one enzyme, butyrate kinase (from butanol-p) or BCoAT (from butanol-CoA), allowing the production of butyrate [52]. Some researchers also reported that *Lachnospiraceae* involve in hydrolyzing glucosides and degrading pectin and cellulose [53]. In our study, all those bacteria, induced by xylitol, were related to SCFAs, especially propionate synthesis.

The concentrations of SCFAs in the gut lumen and mucosa were quantified in vitro. We found that the shift in the SCFAs in CDMN coincided with that in mice. The SCFAs in the mouse feces primarily reflected the SCFAs in the colonic lumen. Acetate and butyrate were mainly distributed in the mucosa (Fig. 3e), indicating that they were responsible for host health by lowering the pH of the mucosa to inhibit the adhesion and proliferation of pathogens. The enrichment of SCFAs, especially propionate and butyrate, is in agreement with the research of Tadashi [8]. Additionally, propionate was used to balance the micro-ecosystem in the lumen. The results of the bacterial composition in the lumen and mucosa supported this phenomenon.

Several studies have also clarified the prebiotic effects of xylitol from various perspectives [7, 8, 10, 11, 26]. In our study, xylitol promoted some bacteria or their corresponding enzymes capable of digesting xylitol, inhibited the growth of *Escherichia*, increased the content of SCFAs, and enhanced the adhesion of *Bifidobacterium*. Tadashi et al. revealed that xylitol promoted the production of propionate and butyrate using human fecal culture and demonstrated that *Anaerostipes hadrus* and *Anaerostipes caccae* augmented butyrate production from xylitol [8]. Tamura et al. suggested that dietary xylitol can affect bone health by enhancing isoflavonoid production via altering the metabolic activity of intestinal microbiota and gut environment [10]. The combined application of *Lactobacillus* and xylitol has been shown to prevent *Clostridium difficile* infection by translocating the colonic mucosa morphology and shifting the gut microbiota [54]. The human study in 1985 provides solid evidence to confirm the prebiotic effects of xylitol on humans [11]. But all of those studies did not tell us how xylitol is utilized and metabolized by microbiota.

Based on results from metatranscriptomic and metabolomic analysis, it could be concluded that the digestion of xylitol was performed by the co-operation of several gut bacteria. Xylitol dehydrogenase (EC 1.1.1.14), xylulokinase (EC 2.7.1.17), and xylulose phosphate isomerase (EC 5.1.3.1) were key enzymes in xylitol metabolism and were present in *Bacteroides* and *Lachnospiraceae*. Phosphate acetyltransferase (EC2.3.1.8), which is a crucial enzyme for propionate synthesis, was mainly distributed in *Bifidobacterium* and *Escherichia* in the presence of xylitol. They all supported the energy metabolism and growth of micro-ecology, but on the other hand, they enhanced the concentration of propionate, which can lower pH to restrict the growth of *Bacteroides* and *Escherichia.* It indicated that xylitol can dynamically balance the relative amount of the gut microbiome. This kind of interaction of microbiota that coexists in nature has a profound role in human health. Three kinds of inter-species interactions are defined as beneficial, neutral, or harmful effects on gut-ecology. Regarding recent studies, a novel graph emerged, which emphasizes convoluted networks and inter-dependencies of microbiomes. Those microbiomes are not limited to exchanges of electron donors for growth, but include the exchanges of vitamin, amino acids, siderophores, and other cofactors [50]. Monosaccharides, such as xylitol, xylulose, and 5-P-xylulose, are absorbed by microorganisms through a reversible transport way [55]. High concentrations of xylulose and 5-P-xylulose can be transported outside the cell as a substrate for other microorganisms. Studies have shown that xylitol dehydrogenase (EC 1.1.1.14) is a dehydrogenase, which uses NADP + (H) as a coenzyme [56, 57]. Catalytic dehydrogenation can produce a large amount of NADPH and H+ [58], which can be used for the synthesis of nucleic acids, fatty acids, and ATP to maintain the normal biological activities of microorganisms. Thus, interacting partners of microorganisms in the cross-feeding system use xylitol not only to supply electron donor exchange but also to provide suitable carbon sources and nutrition. In order to further understand the function of xylitol, single-strain bacterial cultivation and co-cultivation experiments were conducted. In the experiments of single-strain bacterial cultivation and co-cultivation, the usage of xylitol by *L. reuteri* and *E. coli* were different (Fig. 4C, D). The cross-feeding, a relationship in which one organism consumes metabolites excreted by another, is a ubiquitous feature of natural microbial communities and can be a key factor in creating complex communities and promoting diversity in environments [59]. The co-cultivation experiments revealed a cross-feeding relationship among *L. reuteri*, *E. coli*, and *B. fragilis*. In addition, from the molecular analysis, we found that xylitol dehydrogenase (EC 1.1.1.14) was a core enzyme that converts xylitol. Interestingly, xylitol dehydrogenase (EC 1.1.1.14) is not distributed in *E. coli*, although *E. coli* is able to use xylitol [58]. In our study, the survival bacteria depend to some extent on the whole microbiome to guarantee carbon flow and exchange its by-products. A study using a gnotobiotic mouse model also demonstrated that extracellular digestion of inulin increased the growth rate of *Bacteroides ovatus*, the by-products of which from inulin catabolism were utilized by primary fermenters such as *Faecalibacterium prausnitzii* and *Bacteroides vulgatus* [60]. The sequential action of a serial of gut microbiota involving fermentation pathways generates the metabolic input for a diverse set of gut microbiota. In addition, electron donor exchanges also contributed to explaining the dynamic interactions [11, 54, 58].

In nature, xylitol, as an intermediate product converted from D-xylose to xylulose, is found in fruits, vegetables, mushrooms, seaweed, and yeast. Different species of yeast are able to ferment xylose to xylitol or ethanol at low oxygen levels with redox imbalance. In *Debaryomyces hansenii*, the low ratio of NAD/NADH in the oxidation step enables the production of xylitol. *Pichia stipites*, with xylose reductase, can generate NAD+ to convert xylitol to ethanol [60]. But in our study, the low concentrations of fungi and yeast did not make a big contribution to xylitol digestion.

On the other hand, due to the spatial difference for microbiota between lumen and mucosa, we found that microbiota in the mucosa was varied with that in the lumen. The spatial structure of microbiota is a determining factor for cooperation and can drive the dynamics of microbiota [31, 58, 59]. Researchers had also reported that successful growth of the co-culture experiments of *Bacillus subtilis* and the vitamin B_1_ auxotroph *Serendipita indica* was achieved only when these microorganisms were cultivated in a spatially organized environment that provides optimal conditions for cooperative interactions [31, 60]. In our study, the cross-feeding of xylitol was a benefit interaction not only for microbiota, but also for the host. In addition, we found that the xylitol obviously inhibited the biofilm formation and the adhesion to mucosa of toxin-producing *E. coli* but had no effects on beneficial bacteria like *B. adolescentism* (unpublished data). This can be a potential mechanism for prebiotic effects of xylitol.

In conclusion, we confirmed the effect of xylitol on propionate synthesis by combining the results of in vivo and in vitro experiments. The results of the metatranscriptome and metabolome provided macro-insights into propionate synthesis. At the same time, the potential mechanism of propionate synthesis was illustrated at the molecular level. In the food industry, we believe that xylitol can be used as a regulator to modulate and balance gut micro-ecology. The healthy and nutritional evaluation of functional foods should be performed on the basis of gut microbiota. We also proposed that the human gut is not only a large and complex fermentation system, but also an ecosystem.

## Conclusion

**Fig. 6:**
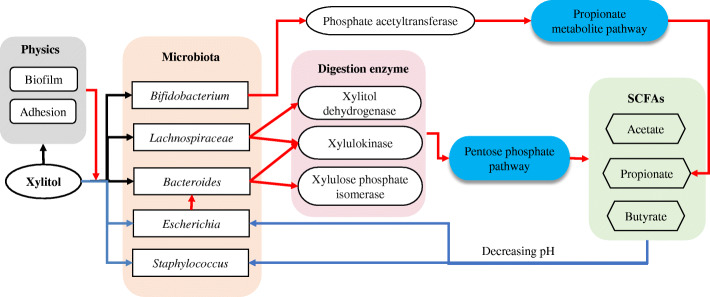
The overall mechanism of xylitol prebiotic effect (red line—promote, blue line—inhibit, black line—neutral)

In this study, the effect of xylitol on the gut microbiota and metabolic activities of mice was investigated by feeding mice xylitol. With the in vitro colon simulation system, the effects of xylitol on the dynamic changes in the microorganisms in the ascending, transverse, and descending colon and its effects on gas and SCFAs production were studied. Key bacteria were selected for single cultivation and co-culture of two and three bacteria, and the pathway in which xylitol was used by microorganisms was investigated. The effects of xylitol on the regulation of microorganisms were also studied at the molecular level. The possible mechanism of xylitol for propionate synthesis was also explained. The mechanism can be concluded as follows (Fig. 6): xylitol maintained the relative amount of *Bifidobacterium*, *Bacteroides*, *Lactobacillus*, and *Lachnospariace* in vivo and promoted enzymes related to xylitol from those bacteria. Moreover, it increased all SCFAs, especially propionate, in the lumen and butyrate in the mucosa, with a shift in its corresponding bacteria in the gut in vitro. A cross-feeding relationship was observed among *L. reuteri*, *B. fragilis*, and *E. coli* in the utilization of xylitol. At the molecular level, we also revealed a cross-feeding relationship in the microbiome. Xylitol dehydrogenase (EC 1.1.1.14), xylulokinase (EC 2.7.1.17), and xylulose phosphate isomerase (EC 5.1.3.1) were key enzymes in xylitol metabolism and were present in *Bacteroides* and *Lachnospiraceae*. Therefore, they were considered fundamental bacteria in xylitol digestion. In addition, xylitol also affected the metabolic pathway of propionate, significantly promoting the transcriptional level of the propionate rate-limiting enzyme, phosphate acetyltransferase (EC 2.3.1.8), in *Bifidobacterium*. Only those enzymes engaged from different bacteria together can support the energy metabolism and growth of micro-ecology, but on the other hand, they also enhanced the content of propionate, which lowered pH to restrict the growth of *Escherichia.* Through the cross-feeding and competition between those cells, xylitol can dynamically balance the relative amount of gut microbiome, in order to promote the health of the host.

## Methods and materials

### Animals

Twenty-four male C57BL/6 wild-type mice were purchased from Slack Company (Shanghai China). The rats were housed under controlled environmental conditions (temperature 23 ± 1 °C; humidity 55 ± 5%; 12-h light/dark cycle) to adapt for 2 weeks, with a commercial food diet and water freely available. Animal experiments were carried out according to institutional guidelines for the care and the use of laboratory animals and approved by the animal ethics committee of China national institute of occupational health and poison control.

### Xylitol treatment

After the adaption period, the C57BL/6 mice were randomly divided into 3 groups; 8 mice in each group were randomly divided into 3 cages and raised for 3 months. The control group was fed with AIN93M, while the experimental groups were fed with AIN93M supplemented with 2% or 5% xylitol (w/w), i.e., 2.17 g/kg/day or 5.42 g/kg/day, respectively. According to the FDA introduction of clinical trials (Guidance for Industry Estimating the Maximum Safe Starting Dose in Initial Clinical Trials for Therapeutics in Adult Healthy Volunteers), xylitol supplementation is equal to 0.18 g/kg/day and 0.44 g/kg/day for a human being, which means estimated 11 g per day and 26 g per day for a 60-kg adult. Water was freely available. Mice were weighed at day 0 and then monthly until sacrifice. Feces were collected monthly and were stored at − 80 °C until DNA extraction and SCFA determination. After the mice were sacrificed, the brain, pancreas, colon, and liver of each animal were weighed by analytical balance, and the colon length was measured.

### In vitro three-stage colon simulation

In vitro colon fermentation was conducted in Changdao Moni simulation system (CDMN) at Zhejiang Gongshang University as we described previously with some modification [61]. Three vessels (simulating ascending colon, transverse colon, descending colon) with volumes 300 mL, 400 mL, and 300 mL are connected. Fecal microorganisms were inoculated into three vessels with 10% of vessel volume. Each vessel was then supplied with 15 mucosal beads (2 g agar was dissolved in 100 mL of distilled water by heating. After the solution was clear and transparent, it cooled to 60 ± 5 °C. Weigh 0.5 g of mucin to dissolve and adjust the pH to about 6.8. After exposure to UV for 30 min, 7–8-mm mucosal beads were obtained in the spherical abrasive tool). CDMN was automatically supplemented with 0.5 mol/L NaOH solution and diluted HCl to adjust the pH. The temperature was kept at 37 °C with the heating and cooling system. Anaerobic conditions were generated by flushing the headspace of all reactions and medium vessels with N_2_ for 30 min three times per day. After 24 h of inoculation and culture, in order to maintain the normal growth of microorganisms, feed and drain 300 mL of feed was supplied with the same volume waste to maintain the fermentation volume. Three mucosal beads in the vessels were replaced by new ones every day to simulate the mucosal regeneration of gut microbiota. After continuous inoculation for about 1 week, the microorganisms in the fermenter will stabilize (stage 1). Sterile xylitol was added to the feed, and it was ingested at a final concentration of 3% (equal to 0.27 g/kg/day for a human being) to simulate xylitol digestion, which was calculated according to Livesey et al. [23] and Poeker et al. [39]. As 60% of xylitol can enter the colon, 3% of supplementation of xylitol into the simulation system is calculated for an estimated daily intake of 0.27 g/kg/day for a 60-kg adult (estimated 16 g/day), accounting for two reactors volume of 0.7 L compared to 0.75 L for the proximal colon volume (ascending colon, transverse colon), giving a mean retention time of 24 h. The fermentation was stopped after 7 days (stage 2). Collect the fermentation broth and mucosal beads of each colon at each day of stage 2 and store the samples at 4 °C for use.

### DNA extraction and PCR amplification

Bacterial DNA was extracted from 0.3-g feces, 1-mL fermentation broth, and one bead by using the TIANamp Bacteria DNA Kit (DP302-02, TIANGEN) according to the manufacturer’s protocols. The V4-V5 region of the bacterial 16S ribosomal RNA gene was amplified by PCR (95 °C for 2 min; followed by 25 cycles at 95 °C for 30 s, 55 °C for 30 s, and 72 °C for 30 s; and a final extension at 72 °C for 5 min) using primers 338F 5′-ACTCCTACGGGAGGCAGCA-3′ and 806R 5′-GGACTACHVGGGTWTCTAAT-3′. PCRs were performed in a 20-μL mixture in triplicate and contained 4 μL of 5 × FastPfu Buffer, 2 μL of 2.5 mM dNTPs, 0.8 μL of each primer (5 μM), 0.4 μL of FastPfu Polymerase, and 10 ng of template DNA.

Fungal DNA was extracted from 1 mL broth using the Plant Genomic DNA Kit (DP305-02, TIANGEN) according to the manufacturer’s protocols. The ITS2 region of the fungal ITS gene was amplified by PCR (98 °C for 30 s; followed by 35 cycles at 98 °C for 10 s, 54 °C for 30 s, and 72 °C for 450 s; and a final extension at 72 °C for 10 min) using primers fITS7: 5′-GTGARTCATCGAATCTTTG-3′ and ITS4: 5′-TCCTCCGCTTATTGATATGC-3′. PCRs were performed in a 25-μL mixture in triplicate and contained 12.5 μL of 2× Phusion® Hot Start Flex Master Mix, 2.5 μL of each primer (1 μM), and 10 ng of template DNA.

### Illumina MiSeq sequencing

Amplicons were extracted from 2% agarose gels and purified using the AxyPrep DNA Gel Extraction Kit (Axygen Biosciences, Union City, CA, USA) according to the manufacturer’s instructions and quantified using QuantiFluor™ -ST (Promega, USA). Purified amplicons were pooled in equimolar proportions and paired-end sequenced (2 × 300) on an Illumina MiSeq at Mingke Biotechnology (Hangzhou) Co., Ltd., China. The raw reads were deposited into the NCBI Sequence Read Archive (SRA) database.

### Processing of Illumina MiSeq sequencings data

Raw fastq files were demultiplexed and quality-filtered using QIIME (version 1.17) with the following criteria: (i) the 250-bp reads were truncated at any site receiving an average quality score < 20 over a 10-bp sliding window, discarding the truncated reads that were shorter than 50 bp; (ii) exact barcode matches, 2 nucleotide mismatch in primer matches, and reads containing ambiguous bases were removed; and (iii) only sequences with overlap longer than 10 bp were assembled according to their overlap sequence. Reads that could not be assembled were discarded.

Operational units (OTUs) were clustered with a 97% similarity cut-off using UPARSE (version 7.1 http://drive5.com/uparse/), and chimeric sequences were identified and removed using UCHIME. The phylogenetic affiliation of each 16S rRNA gene sequence was analyzed by RDP Classifier (http://rdp.cme.msu.edu/) against the Silva (SSU115) 16S rRNA database using a confidence threshold of 70%. The phylogenetic affiliation of each ITS2 gene was analyzed by RDP Classifier (http://rdp.cme.msu.edu/) against the Unite database.

### Determination of SCFAs

0.3-g sample of pretreated feces from rats (supernatant from the mixture of feces after grinding with 1 mL ultra-pure water after centrifugation at 6000 rpm for 10 min) or 1 mL of fermentation broth mixed with 100 μL concentrated hydrochloric acid and 5 mL of ethyl ether was used. After extraction at room temperature for 20 min, the mixture was centrifuged at 5000 rpm for 10 min at 4 °C. The top layer was removed to clean the tube and mixed with 500 μL of 1 M NaOH. After the second extraction and centrifugation, the water phase in the bottom layer was collected and removed to another tube. The bottom layer was filtered with a 0.22-μm filter after adding 100 μL concentrated hydrochloric acid. The SCFAs in the aliquot were determined by HPLC. Column: ZORBAX SB-Aq (4.6 × 250 mm 5-Micron); mobile phase 0.025% phosphoric acid solution (pH = 2.8): acetonitrile = 95:5, flow rate 1.0 mL/min; injection volume 20 μL; detection wavelength 210 nm; column temperature 30 °C [62].

### Metatranscriptome sequencing

#### RNA isolation, purification, and quantification

Total RNA was isolated and purified using TRIzol reagent (Invitrogen, Carlsbad, CA, USA) following the manufacturer's procedure. The RNA amount and purity of each sample were quantified using a NanoDrop ND-1000 (NanoDrop, Wilmington, DE, USA). The RNA integrity was assessed by Agilent 2100 with a RIN number > 7.0.

#### cDNA library construction

Approximately 0.5 g of total RNA was used to deplete ribosomal RNA according to the instructions of the Ribo-Zero™ rRNA Removal Kit (Illumina, San Diego, USA). After removing ribosomal RNAs, the remaining RNAs were fragmented into small pieces using divalent cations under high temperatures. Then, the cleaved RNA fragments were reverse-transcribed to create cDNA, which was then used to synthesize U-labeled second-stranded DNAs with *E. coli* DNA polymerase I, RNase H, and dUTP. An A-base was then added to the blunt ends of each strand, preparing them for ligation to the indexed adapters. Each adapter contained a T-base overhang for ligating the adapter to the A-tailed fragmented DNA. Single- or dual-index adapters were ligated to the fragments, and size selection was performed with AMPureXP beads. After heat-labile UDG enzyme treatment of the U-labeled second-stranded DNAs, the ligated products were amplified by PCR under the following conditions: initial denaturation at 95 °C for 3 min; 8 cycles of denaturation at 98 °C for 15 s, annealing at 60 °C for 15 s, and extension at 72 °C for 30 s; and a final extension at 72 °C for 5 min. The average insert size for the final cDNA library was 300 bp (± 50 bp). Finally, we performed 150-bp paired-end sequencing on an Illumina HiSeq 4000 (LC-Bio Technology Co., Ltd., Hangzhou, Zhejiang Province, China) following the vendor's recommended protocol.

#### Processing of metatranscriptome sequencing data

Raw sequencing reads were processed to obtain valid reads for further analysis. First, sequencing adapters were removed from sequencing reads using cutadapt v1.9. Second, low-quality reads were trimmed by fqtrim v0.95 using a sliding-window algorithm. Third, reads were aligned to the host genome using bowtie2 v2.2.0 to remove host contamination. Once quality-filtered reads were obtained, they were de novo assembled to construct the metatranscriptome for each sample with Trinity v2.2.0. All metatranscriptome contigs of all samples were clustered by CD-HIT v4.6.1 to obtain unigenes. Unigene abundance for a certain sample was estimated by TPM based on the number of aligned reads by bowtie2 v2.2.0. The lowest common ancestor taxonomy of unigenes was obtained by aligning them against the NCBI NR database by DIAMOND v 0.7.12. Similarly, the functional annotation (GO, KEGG, eggNOG, CAZy, CARD, PHI) of unigenes was obtained. Based on the taxonomic and functional annotation of unigenes and the abundance profile of unigenes, the differential analysis was carried out at each taxonomic, functional, or gene-wise level by Fisher’s exact test (non-replicated groups) or Kruskal–Wallis test (replicated groups).

#### Metabolomic profiling identification

The collected samples were thawed on ice, and metabolites were extracted with 50% methanol. Briefly, 20 μL of a sample was extracted with 120 μL of precooled 50% methanol, vortexed for 1 min, and incubated at room temperature for 10 min; the extraction mixture was then stored overnight at − 20 °C. After centrifugation at 4000*g* for 20 min, the supernatants were transferred into new 96-well plates. The samples were stored at − 80 °C prior to the LC-MS analysis. In addition, pooled QC samples were also prepared by combining 10 μL of each extraction mixture.

All samples were acquired by the LC-MS system following the instructions of the instrument. First, all chromatographic separations were performed using an ultra-performance liquid chromatography (UPLC) system (SCIEX, UK). An ACQUITY UPLC T3 column (100 mm × 2.1 mm, 1.8 μm, Waters, UK) was used for the reversed-phase separation. The column oven was maintained at 35 °C. The flow rate was 0.4 mL/min, and the mobile phase consisted of solvent A (water, 0.1% formic acid) and solvent B (acetonitrile, 0.1% formic acid). The gradient elution conditions were set as follows: 0~0.5 min, 5% B; 0.5~7 min, 5 to 100% B; 7~8 min, 100% B; 8~8.1 min, 100 to 5% B; 8.1~10 min, 5% B. The injection volume for each sample was 4 μL.

A high-resolution tandem mass spectrometer TripleTOF5600plus (SCIEX, UK) was used to detect metabolites eluted from the column. The Q-TOF was operated in both positive and negative ion modes. The curtain gas was set at 30 PSI, the ion source gas1 was set at 60 PSI, the ion source gas2 was set at 60 PSI, and the interface heater temperature was 650 °C. In the positive ion mode, the ion spray voltage floating was set at 5000 V. In the negative ion mode, the ions pray voltage floating was set at − 4500 V. The mass spectrometry data were acquired in IDA mode. The TOF mass range was from 60 to 1200 Da. The survey scans were acquired in 150 ms, and as many as 12 product ion scans were collected if a threshold of 100 counts per second (counts/s) was exceeded and a 1+ charge-state was detected. The total cycle time was fixed to 0.56 s. Four times bins were summed for each scan at a pulse frequency value of 11 kHz through monitoring of the 40-GHz multichannel TDC detector with four-anode/channel detection. Dynamic exclusion was set at 4 s. During the acquisition, the mass accuracy was calibrated every 20 samples. Furthermore, to evaluate the stability of the LC-MS during the whole acquisition, a quality control sample (pool of all samples) was acquired after every 10 samples.

The acquired MS data pretreatments including peak picking, peak grouping, retention time correction, second peak grouping, and annotation of isotopes and adducts were performed using XCMS software. LC–MS raw data files were converted into mzXML format and then processed by the XCMS, CAMERA, and metaX toolbox implemented with the R software. Each ion was identified by combining retention time (RT) and *m*/*z* data. Intensities of each peak were recorded and a three-dimensional matrix containing arbitrarily assigned peak indices (retention time-*m*/*z* pairs), sample names (observations), and ion intensity information (variables) was generated.

The online KEGG, HMDB database was used to annotate the metabolites by matching the exact molecular mass data (*m*/*z*) of samples with those from the database. If a mass difference between observed and the database value was less than 10 ppm, the metabolite would be annotated and the molecular formula of metabolites would further be identified and validated by the isotopic distribution measurements. We also used in-house fragment spectrum library of metabolites to validate the metabolite identification.

The intensity of peak data was further pre-processed by metaX. Those features that were detected in less than 50% of QC samples or 80% of biological samples were removed, the remaining peaks with missing values were imputed with the k-nearest neighbor algorithm to further improve the data quality. PCA was performed for outlier detection and batch effects evaluation using the pre-processed dataset. Quality control-based robust LOESS signal correction was fitted to the QC data with respect to the order of injection to minimize signal intensity drift over time. In addition, the relative standard deviations of the metabolic features were calculated across all QC samples, and those > 30% were then removed.

Student *t* tests were conducted to detect differences in metabolite concentrations between 2 phenotypes. The *p* value was adjusted for multiple tests using an FDR (Benjamini–Hochberg).

Supervised PLS-DA was conducted through metaX to discriminate the different variables between groups. The VIP value was calculated. A VIP cut-off value of 1.0 was used to select important features.

### Cultivation of single, two, and three bacteria to evaluate xylitol fermentation

#### Bacterial cultivation

*Saccharomyces cerevisiae* S33 was inoculated into Yeast Peptone Dextrose medium (Hangzhou microbial reagents Co. LTD, China), *Bifidobacterium adolescentis* JYBA-16 and *Lactobacillus reuteri* DSM20058 were inoculated into MRS medium (Hangzhou microbial reagents Co. LTD, China), and *Bacteroides fragilis* BNCC 336948 and *Escherichia coli* CICC 10032 were inoculated in TSB and LB medium (Hangzhou microbial reagents Co. LTD, China), respectively. After growing, aliquots from each medium were inoculated on the corresponding agar medium to pick pure and single colonies. The single and pure colonies were pre-cultured into the corresponding medium for growth until OD_600_ reaches 0.6~0.8. One hundred microliters of each pure bacterial suspension was cultured to 10-mL GAM medium at 37 °C until OD_600_ reaches 0.6~0.8. GAM contains (/L) 10 g pancreatic casein peptone, 3 g soy protein, 5 g yeast extract, 2 g beef extract, 13.5 g digested serum, 1.2 g beef liver extract, 2.5 g potassium dihydrogen phosphate, 3 g sodium chloride, 0.3 g cysteine-HCl, 0.15 g sodium thioglycolate, 2 g glucose, and 0.3 g soluble starch. The pH was adjusted to 7.3 ± 0.1. *B.adolescentis*, *L.reuteri*, and *B.fragilis* were incubated into anaerobic condition, while *S. cerevisiae* and *E.coli* grown aerobically with 150 rpm shaking.

A total of 100 μL of bacterial suspension from the cultured GAM medium was added to the fresh GAM medium, and the number of bacteria was counted every 6 h until the concentration reached 10^9^ cell/mL by hemocytometer. Then, the suspension was diluted to 1 × 10^9^ cell/mL by saline to guarantee the concentration of each bacterial inoculum for xylitol fermentation is the same.

#### Xylitol fermentation

The media of GAM with glucose and soluble starch depletion are named non-carbohydrate GAM media. The composition of GAM + glucose, GAM + xylitol, and combination of GAM media are non-carbohydrate GAM media with respective addition of 20 g/L glucose, 20 g/L xylitol, and 10 g/L xylitol + 10 g/L glucose. A total of 100 μL of each diluted bacterial suspension from GAM medium (the concentration of diluted bacterial suspension was 1 × 10^9^ cell/mL) was inoculated at 100 mL of GAM + glucose medium, GAM + xylitol medium, a combination of GAM media, and non-carbohydrate GAM medium, for single-strain fermentation.

100 μL of diluted *E. coli* and *L. reuteri* suspension were co-cultured in 100 mL of GAM + glucose and GAM + xylitol media for double-strain fermentation. One hundred microliters of diluted *E. coli*, *L. reuteri*, and *B. fragilis* was transferred to 100 mL of GAM + glucose and GAM + xylitol media for three-strain fermentation. The fermentation lasted for 48~60 h under anaerobic condition. Two milliliters of aliquots from each medium was sampled every 2–4 h and diluted from 10^0^ to 10^−7^ for plating and counting. The sampled fermentation broth of *E. coli*, *L. reuteri*, *B. fragilis*, *S. cerevisiae*, and *B. adolescentis* were respectively plated into Maconkey Agar media, Chalmers Agarmedia, Modified GAM Agar media, Li-Mupirocin and Cysteine hydrochloride modified MRS media, and Yeast Peptone Dextrose Agar media and MRS for enumeration.

Maconkey Agar media, Modified GAM Agar media, and Li-Mupirocin and Cysteine hydrochloride modified MRS media were purchased from Qingdao Haibo Biotechnology Co. LTD, China. Chalmers Agarmedia, Yeast Peptone Dextrose Agar media, and MRS were purchased from Hangzhou microbial reagents Co. LTD, China.

### Statistical analyses

SCFAs and microorganisms were analyzed by CCA (Canoco 5). The correlation between microbes was calculated by Spearman correlation with SPSS, and the correlation coefficient of correlation coefficient greater than 0.2 was selected and plotted with Cytoscape (v3.4.0). All data are shown as the mean ± SD. The data were analyzed with ANOVA and Duncan’s test for multiple comparisons with SPSS ver. 17.0. A value of *p* < 0.05 was considered significant. FDR analysis of Benjamini and Hochberg (BH) method was applied to reduce the false positive rate for the high amount of data and a value of *Q* < 0.05 was considered significant after FDR analysis. The optimal distribution was analyzed by @risk v7.5.0.1.

## Supplementary Information

**Additional file 1.** Additional information.

**Additional file 2: Figure S1.** Gamma distribution of microbiome fitted according to chi-square standard.

**Additional file 3: Figure S2.** Expon distribution of alpha-diversity fitted according to chi-square standard.

**Additional file 4: Figure S3.** Phylum analysis of relative abundance of bacteria in mice colon. (Con: control group; 2%XY-a cohort of mouse supplied with diet contains 2% of xylitol; 5%XY- a cohort of mouse supplied with diet contains 5% of xylitol; the number means the month to feed the mice).

**Additional file 5: Figure S4.** Phylum analysis of relative abundance of bacteria in different parts of in vitro colon (A: bacteria in lumen, B: bacteria in mucosa. AC, TC, DC presented Ascending Colon, Transverse colon and descending colon, the number means the days for adding xylitol).

**Additional file 6: Figure S5.** Histogram of changes in gas proportions in each segment of the colon (AC, TC, DC presented Ascending Colon, Transverse colon and descending colon. The number means the days for adding xylitol).

**Additional file 7: Figure S6.** Effects of xylitol on propanoate metabolites (Red-up regulate, Blue-down regulate).

**Additional file 8: Figure S7.** Effects of xylitol on pentose and glucuronate interconversion metabolites in vitro (Redup regulate, Blue-down regulate).

**Additional file 9: Figure S8.** Effects of xylitol on propionate metabolic enzymes (Red-up regulate, Blue-down regulate).

## Data Availability

The 16S rRNA and ITS gene sequencing dataset generated in this study are stored in National Center for Biotechnology Information (NCBI, https://www.ncbi.nlm.nih.gov/) and the project no. is PRJNA604899, PRJNA604957, PRJNA605597.
