# Supplementary material for: Xylitol enhances synthesis of propionate in the colon via cross-feeding of gut microbiota
Source: Microbiome. 2021 Mar 18;9:62. doi: 10.1186/s40168-021-01029-6 (PMC7977168; doi:10.1186/s40168-021-01029-6)
Supplement: Supplementary file 2 — Additional file 1. Additional information. [file 40168_2021_1029_MOESM1_ESM.docx]

**Additional information**

**Method**

**Determination of biofilms by crystal violet staining**

*E. coli* SPHJ, *B. adolescentis* JYBA-16, *L. reuteria* DSM20058*,* *B. infatns* BNCC 336948 and *S.cerevisiae* S33 were activated using saved methods summarized in xylitol fermentation of single bacteria. The pure colonies were cultured into the corresponding liquid medium for growth until OD_600_ reaching to 0.6. Bacteria in 5 mL of each culture media was harvested by centrifuged at 6000 rpm for 5min. Then cells were washed twice by 5 mL sterilized saline. Afterwards,1 mL suspension was added into 8 mL medium with 0，2%，5% (w/v) xylitol supplementation, respectively. 200 μL homogenized liquid was sampled from each media and transferred to 96-well plates incubating at 37℃. After 36 hours, the liquid was removed and each well was washed by 200 μL sterile PBS buffer for 3 times. 100 μL methanol was moved out after fixing the biofilm for 15 min and dried in room temperature. 100 μL crystal violet solution (1% W/V) was added into each well to stain the biofilm at room temperature for 5 min. 100 μL acetic acid solution (33% V/V) was added and incubated at 37℃ to dissolve crystal violet. The biofilm was detected at OD_590_. The experiments were repeated in triplicate.

**Effect of xylitol on mucin adhesion of *E. coli* SPHJ and *B. adolescentis* JYBA-16**

Mucin(sigma USA) from pig was dissolved in HEPES-Hanks buffer（HH）in 15 mL falcon tube and sterilized under UV for 15 min. The concentration of mucin solution was 50 mg/mL.100 μL mucin solution was added to 96-well plates and fixed at 4℃ overnight, Then HH washed twice to remove the rest mucin. After activation of *E. coli* and *B. adolescentis* as previously described in the method of xylitol fermentation of single bacteria, fresh bacterial cultures were centrifuged at 6000rpm for 5min. Cells were resuspended by HH and adjusted to 2x10^6^ cells/mL by hemocytometer after washed twice by HH. 100 μL pretreated *E. coli* and *B. adolescentis* were solely incubated with 50 μL 0, 10%, 25%(w/v) xylitol solution respectively (xylitol dissolved by PBS and sterilized by 0.22 μm filter) to simulate the mucin adhesion. To simulate competition, resistance and replacement for mucin adhension, three conditions were set in 96-well plates with 0%、2%、5% xylitol. 1) 50 μL pretreated *E. coli* and 50 μL pretreated *B.* *adolescentis* were incubated simultaneously; 2) 50 μL *E. coli* were incubated one hour after 50 μL *B.* *adolescentis* incubation; 3) 50 μL *E. coli* were incubated an hour priority to 50 μL *B.* *adolescentis.* Liquid was removed after an hour incubation. Bacteria were washed by 150 μL by HH for 5 times before elution of attached bacteria by 200 μL PBS (with 1％Triton-100X). Eluent was diluted from 10^-2^ to 10^-4^ for plating and counting. *E. coli* and *B. adolescentis* were respectively plated into Maconkey Agar media and MRS for enumeration. The experiments were repeated in triplicate.

**Effect of xylitol on HT-29 cells adhesion of *E. coli* SPHJ and *B. adolescentis* JYBA-16**

HT-29 cells were passaged in DMEM medium under the condition of 37℃ and 5% CO_2_. The modified DMEM medium was supplied with1% penicillin and streptomycin resistance and 10% fetal bovine serum. HT - 29 cells with limited passaged (passage number: 4~30), were incubated into 8-well plates with a density of 10^4^ ~ 10^5^ / well and cultivated for 24 h until cells covered with pore plate. Then cell was washed out twice by 1 ml sterile PBS. After activation of *E. coli* and *B. adolescentis* as previously described in the method of xylitol fermentation of single bacteria, fresh bacterial cultures were centrifuged at 6000rpm for 10min. Cells were resuspended by DMEM medium without fetal bovine serum and adjusted to 2x10^7^ CFU/mL by hemocytometer after washed twice by sterilized PBS.0.5 mL pretreated *E. coli* and *B. adolescentis* were solely incubated with 0.5 mL 0, 4%, 10%(w/v) xylitol solution (dissolved by PBS and sterilized by 0.22μm filter) at 37℃ for an hour to simulate the adhesion to HT-29 cells. To simulate competition, resistance and replacement for HT-29 cells, three conditions were set in 8-well plates with 0%、2%、5% xylitol. 1) 0.25 mL pretreated *E. coli* and 0.25 mL pretreated *B.* *adolescentis* were incubated simultaneously; 2) 0.25 mL *E. coli* were incubated one hour after 0.25 mL *B.* *adolescentis* incubation; 3) 0.25 mL *E. coli* were incubated an hour priority to 0.25 mL *B.* *adolescentis.* Liquid was removed after an hour incubation. Bacteria were washed by 1mL by PBS gently for 5 times before resuspending of attached bacteria by 0.5 mL 0.25% trypsin-0.02% EDTA. 2 mL complete medium was added to stop digestion, centrifugation at 1500rpm for 5min. The supernatant was removed, and the cells were resuspended with 1mLPBS (with 1% triton-100x), and the cells were lysed for 30 min at room temperature. Lysate diluted from 10^0^ to 10^-4^ for plating and counting. *E. coli* and *B. adolescentis* were respectively plated into Maconkey Agar media and MRS for enumeration. The experiments were repeated in triplicate.

Table S1 Changes of short-chain fatty acid content

| mmol/g | Acetate | Propionate | Butyrate | Valerate | summary |
| --- | --- | --- | --- | --- | --- |
| Con1 | 3.16±0.75 | 0.54±0.12 | 1.51±0.10 | 3.93±0.11 | 9.15±0.30 |
| 2%XY1 | 5.74±1.14^*^ | 2.31±0.42 | 1.68±1.04 | 5.75±0.61 | 15.48±0.44^*^ |
| 5%XY1 | 4.60±0.27^*^ | 3.23±1.06^*^ | 0.64±0.36 | 1.13±0.04 | 9.60±0.35 |
| Con2 | 6.98±3.98 | 2.91±0.25 | 0.05±0.03 | 2.43±0.03 | 12.38±4.24^*^ |
| 2%XY2 | 4.74±0.87 | 2.14±0.27 | 0.07±0.04 | 0.59±0.02 | 7.48±1.09 |
| 5%XY2 | 10.39±0.34 | 10.02±0.30^*^ | 3.06±0.56^*^ | 9.04±0.12^*^ | 32.52±0.47^*^ |
| Con3 | 6.40±1.19 | 2.20±0.15 | 0.02±0.02 | 2.72±0.02 | 11.34±0.17 |
| 2%XY3 | 9.77±1.20 | 6.00±0.60^*^ | 0.08±0.04 | 4.53±0.01 | 20.3±0.68^*^ |
| 5%XY3 | 14.49±0.11^*^ | 10.09±0.05^*^ | 1.14±0.05^*^ | 5.61±0.03^*^ | 31.33±0.16^*^ |

2%XY、5%XY have significant difference compared to Con，**p<*0.05; Con: control group; 2%XY-a cohort of mouse supplied with diet contains 2% xylitol; 5%XY- a cohort of mouse supplied with diet contains 5% xylitol；the number means the month to feed the mice；1,2,3 means the first, second and third month.

Table S2 Electronic nose sensor and gas correspondence list

| Sensor number | Sensor type | Sensitive gas |
| --- | --- | --- |
| SE1 | TGS829 | Ammonia, Amines |
| SE2 | mq-136 | Hydrogen Sulfide, Sulfide |
| SE3 | TGS821 | Hydrogen |
| SE4 | TGS822 | Alcohol, organic solvent |
| SE5 | mq-138 | Alcohols, ketones, aldehydes, aromatic compounds |
| SE6 | mq-4 | Methane, biogas |
| SE7 | TGS813 | Combustible gas |
| SE8 | TGS2602 | VOC (mostly used for environmental gas pollution detection) |
| SE9 | mq-5 | Liquefied gas, natural gas, gas |
| SE10 | TGS2610 | Liquefied gas, combustible gas |
| SE11 | mq-2 | Alkane, alcohol, natural gas, smoke |
| SE12 | TGS2620 | Alcohol, organic solvent |
| S13 | TGS2600 | Smoke, cooking odor |
| SE14 | TGS2611 | Methane, gas |
