# Supplementary figures and images for: Xylitol enhances synthesis of propionate in the colon via cross-feeding of gut microbiota
Source: Microbiome. 2021 Mar 18;9:62. doi: 10.1186/s40168-021-01029-6 (PMC7977168; doi:10.1186/s40168-021-01029-6)

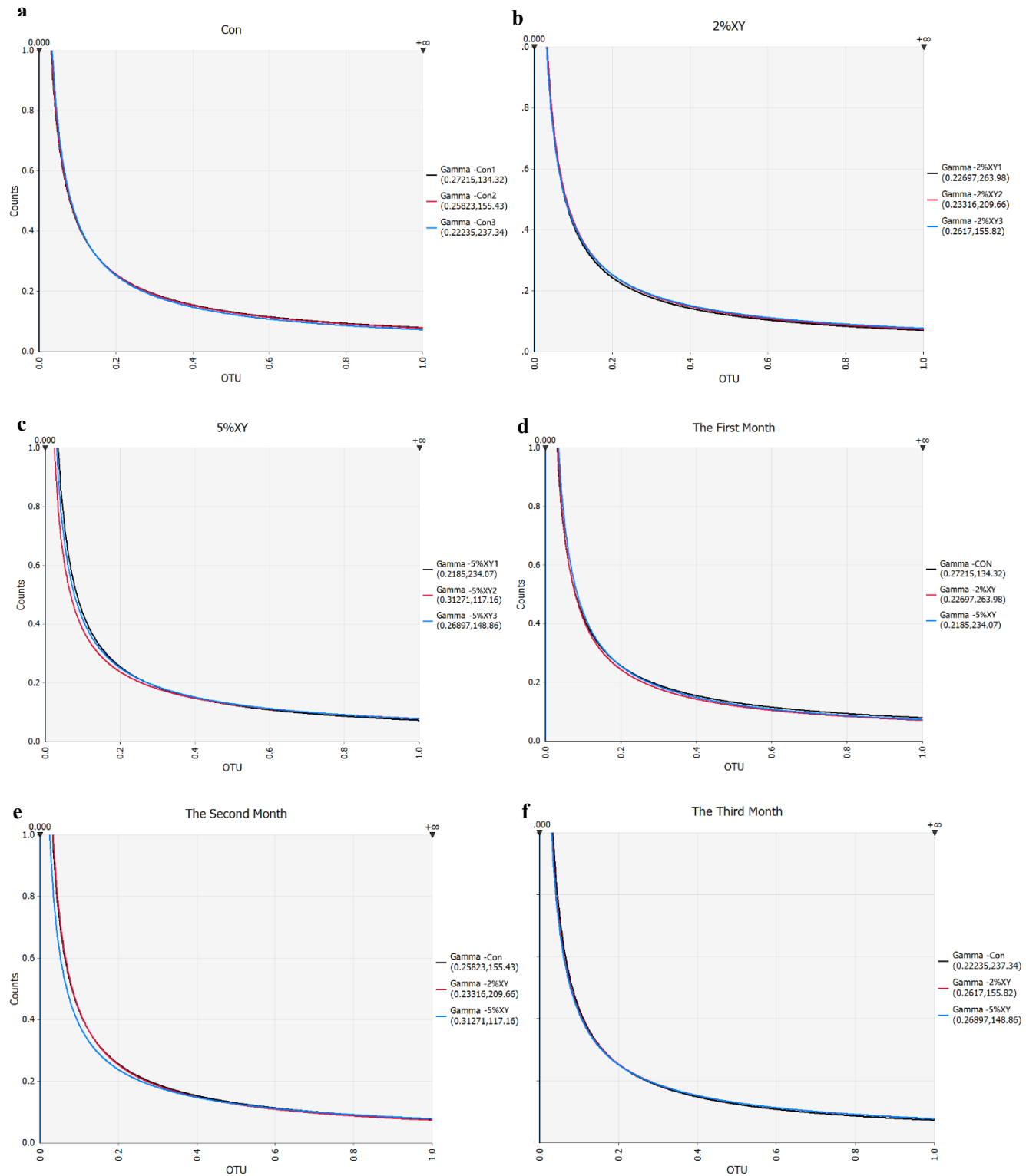

**Figure S1** Gamma distribution of microbiome fitted according to chi-square standard.

Supplement: Supplementary file 3 — Additional file 2: Figure S1. Gamma distribution of microbiome fitted according to chi-square standard. [file 40168_2021_1029_MOESM2_ESM.pdf]

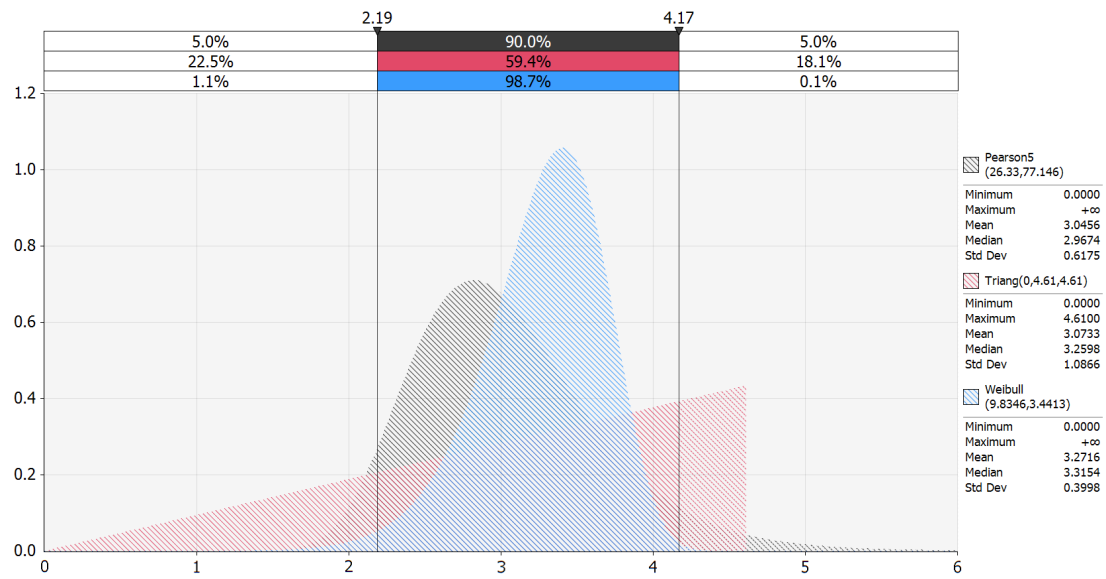

**Figure S2** Expon distribution of alpha-diversity fitted according to chi-square standard.

Supplement: Supplementary file 4 — Additional file 3: Figure S2. Expon distribution of alpha-diversity fitted according to chi-square standard. [file 40168_2021_1029_MOESM3_ESM.pdf]
