# Supplementary material for: Xylitol enhances synthesis of propionate in the colon via cross-feeding of gut microbiota
Source: Microbiome. 2021 Mar 18;9:62. doi: 10.1186/s40168-021-01029-6 (PMC7977168; doi:10.1186/s40168-021-01029-6)
Supplement: Supplementary file 5 — Additional file 4: Figure S3. Phylum analysis of relative abundance of bacteria in mice colon. (Con: control group; 2%XY-a cohort of mouse supplied with diet contains 2% of xylitol; 5%XY- a cohort of mouse supplied with diet contains 5% of xylitol; the number means the month to feed the mice). [file 40168_2021_1029_MOESM4_ESM.pdf]

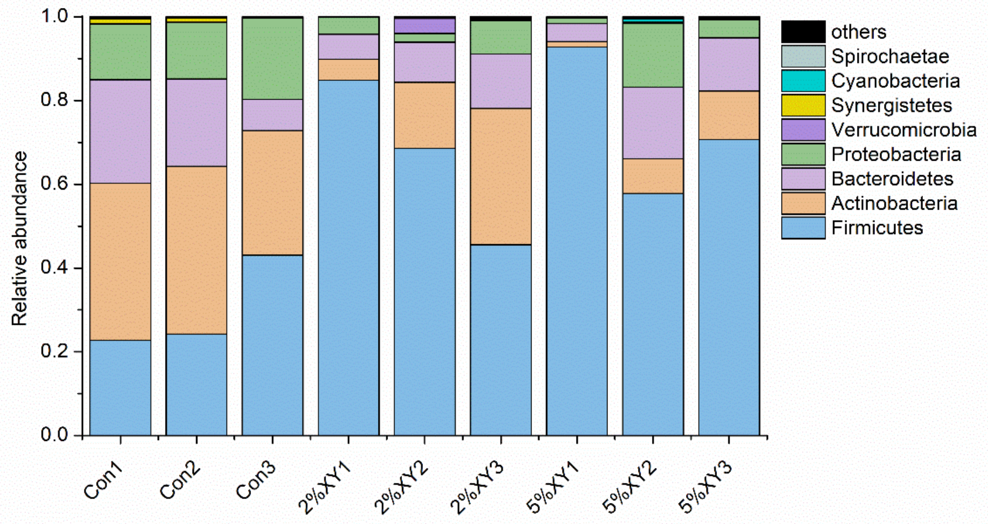

Figure S3 Phylum analysis of relative abundance of bacteria in mice colon. (Con: control group; 2%XY-a cohort of mouse supplied with diet contains 2% of xylitol; 5%XY- a cohort of mouse supplied with diet contains 5% of xylitol； the number means the month to feed the mice).
