# Supplementary material for: Xylitol enhances synthesis of propionate in the colon via cross-feeding of gut microbiota
Source: Microbiome. 2021 Mar 18;9:62. doi: 10.1186/s40168-021-01029-6 (PMC7977168; doi:10.1186/s40168-021-01029-6)
Supplement: Supplementary file 6 — Additional file 5: Figure S4. Phylum analysis of relative abundance of bacteria in different parts of in vitro colon (A: bacteria in lumen, B: bacteria in mucosa. AC, TC, DC presented Ascending Colon, Transverse colon and descending colon, the number means the days for adding xylitol). [file 40168_2021_1029_MOESM5_ESM.pdf]

A

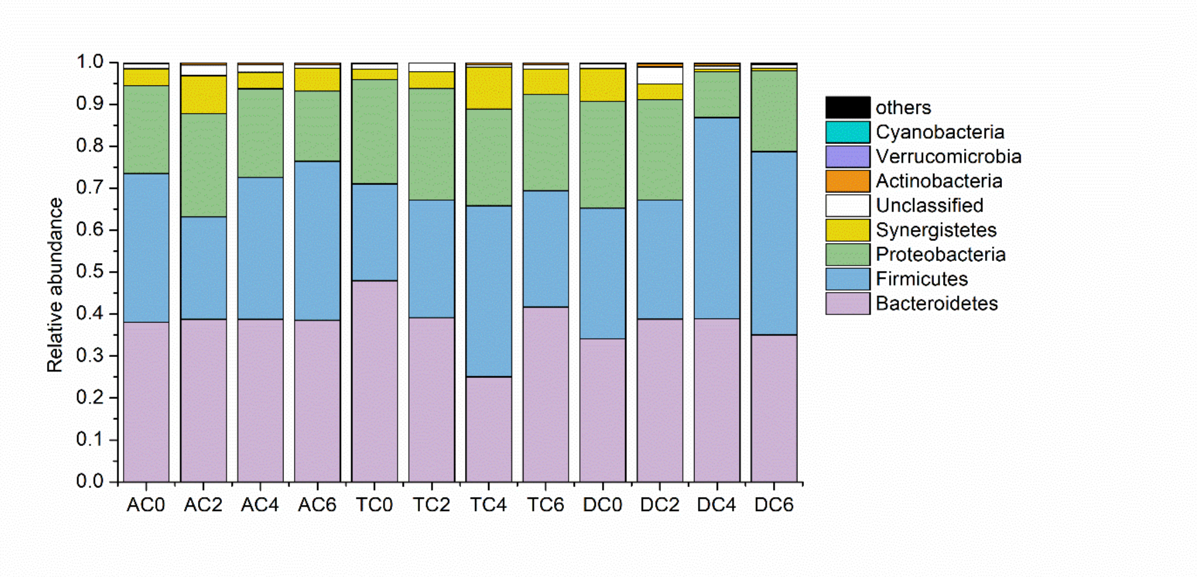

B

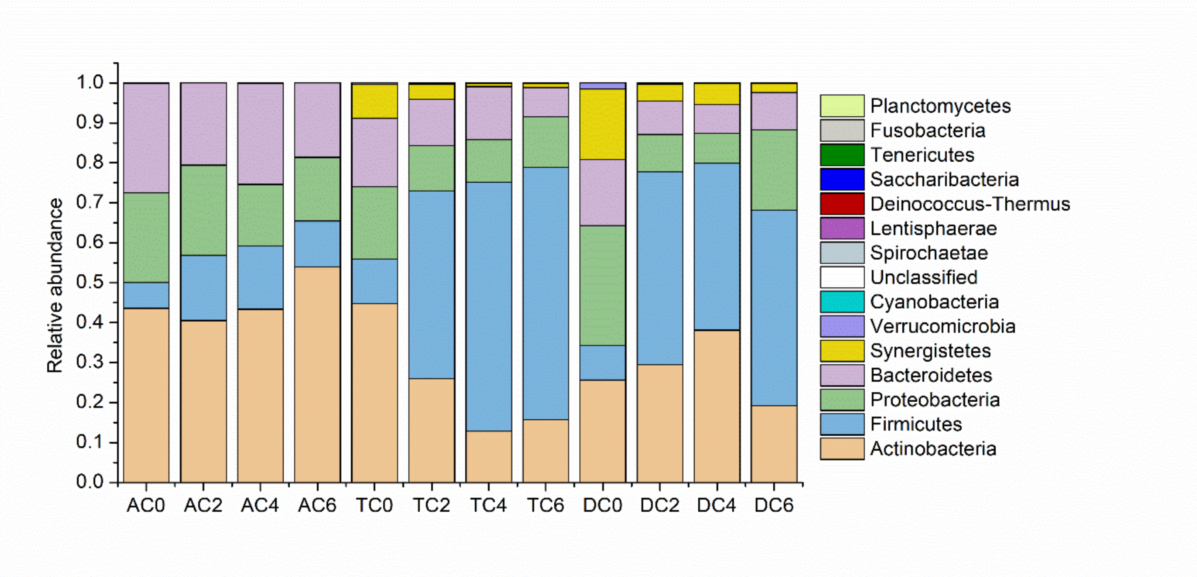

Figure S4 Phylum analysis of relative abundance of bacteria in different parts of *in vitro* colon (A: bacteria in lumen, B: bacteria in mucosa. AC, TC, DC presented Ascending Colon, Transverse colon and descending colon, the number means the days for adding xylitol).
