# Supplementary material for: Xylitol enhances synthesis of propionate in the colon via cross-feeding of gut microbiota
Source: Microbiome. 2021 Mar 18;9:62. doi: 10.1186/s40168-021-01029-6 (PMC7977168; doi:10.1186/s40168-021-01029-6)
Supplement: Supplementary file 7 — Additional file 6: Figure S5. Histogram of changes in gas proportions in each segment of the colon (AC, TC, DC presented Ascending Colon, Transverse colon and descending colon. The number means the days for adding xylitol). [file 40168_2021_1029_MOESM6_ESM.pdf]

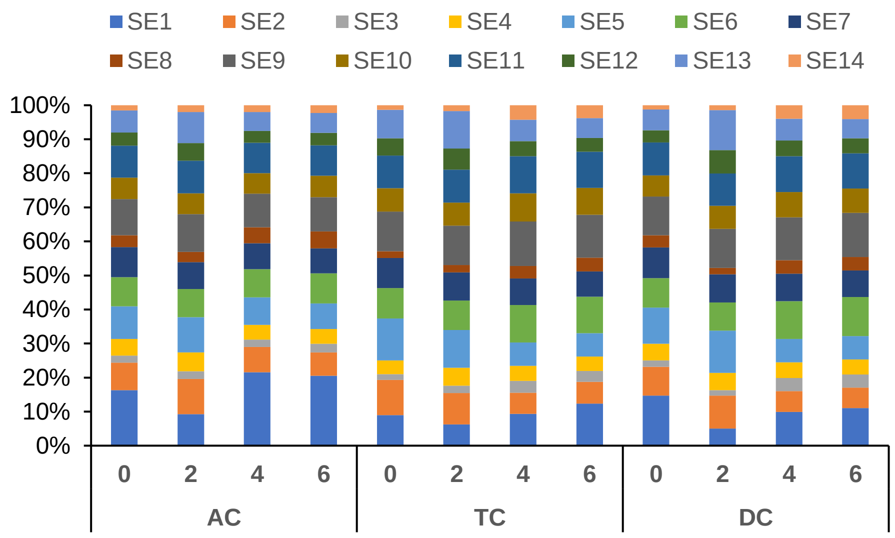

Figure S5 Histogram of changes in gas proportions in each segment of the colon (AC, TC, DC presented Ascending Colon, Transverse colon and descending colon. The number means the days for adding xylitol).
