# Supplementary material for: Xylitol enhances synthesis of propionate in the colon via cross-feeding of gut microbiota
Source: Microbiome. 2021 Mar 18;9:62. doi: 10.1186/s40168-021-01029-6 (PMC7977168; doi:10.1186/s40168-021-01029-6)
Supplement: Supplementary file 9 — Additional file 8: Figure S7. Effects of xylitol on pentose and glucuronate interconversion metabolites in vitro (Redup regulate, Blue-down regulate). [file 40168_2021_1029_MOESM8_ESM.pdf]

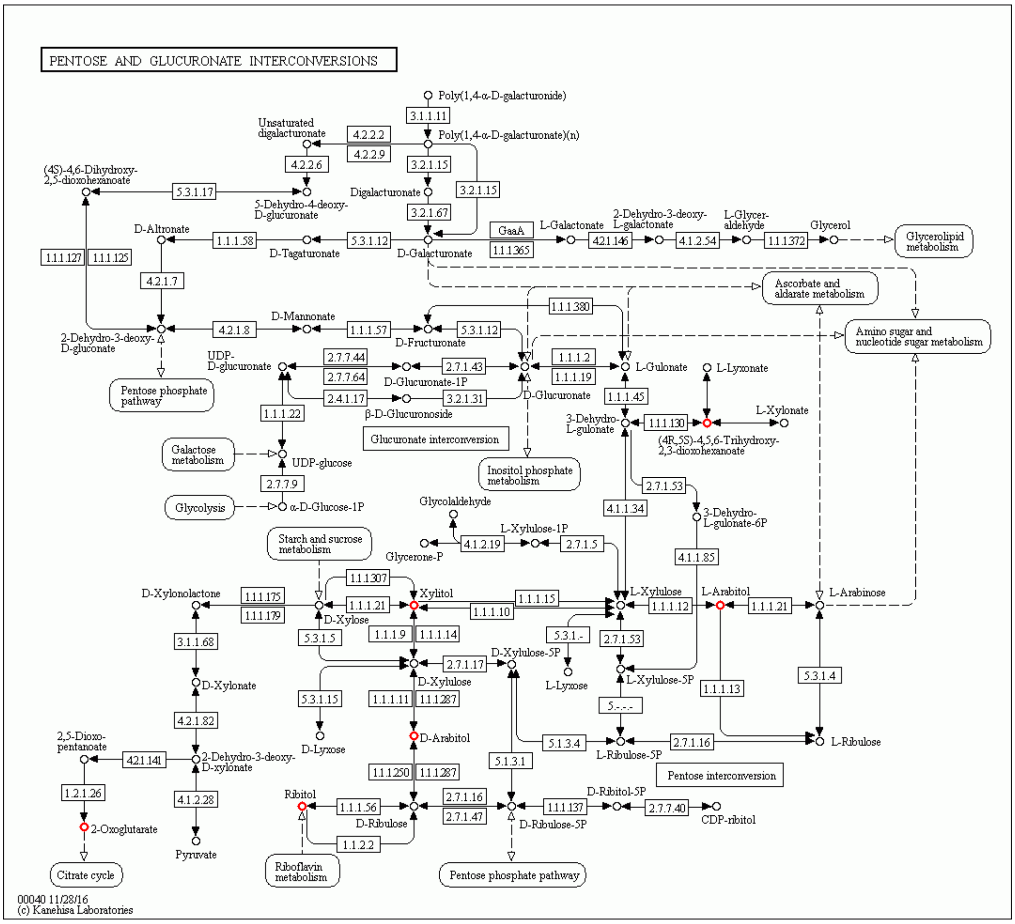

Figure S7 Effects of xylitol on pentose and glucuronate interconversion metabolites *in vitro* (Red-up regulate, Blue-down regulate).
