# Supplementary material for: Xylitol enhances synthesis of propionate in the colon via cross-feeding of gut microbiota
Source: Microbiome. 2021 Mar 18;9:62. doi: 10.1186/s40168-021-01029-6 (PMC7977168; doi:10.1186/s40168-021-01029-6)
Supplement: Supplementary file 8 — Additional file 7: Figure S6. Effects of xylitol on propanoate metabolites (Red-up regulate, Blue-down regulate). [file 40168_2021_1029_MOESM7_ESM.pdf]

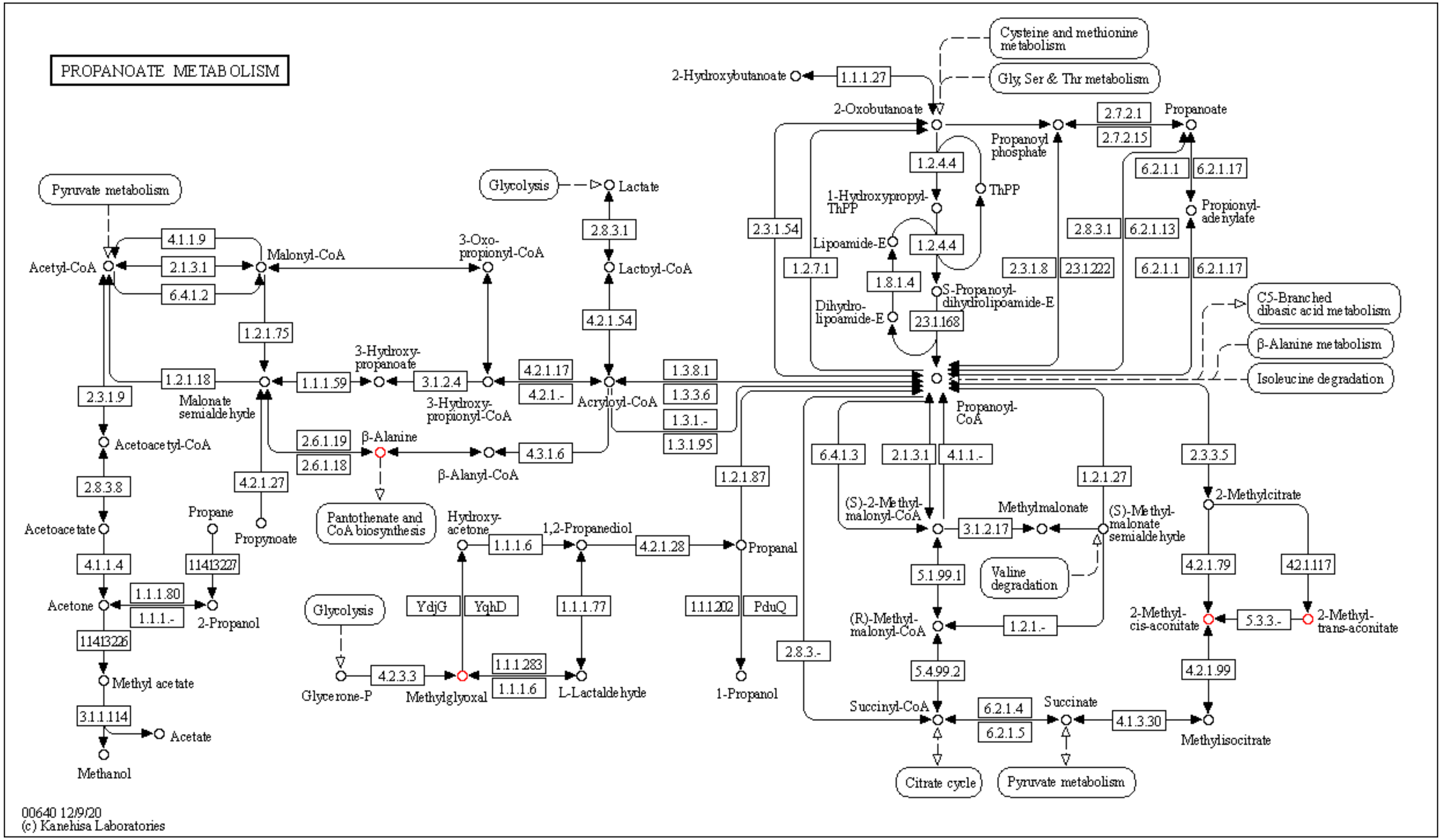

Figure S6 Effects of xylitol on propanoate metabolites (Red-up regulate, Blue-down regulate).
